# A humanized IL-2 mutein expands Tregs and prolongs transplant survival in preclinical models

**DOI:** 10.1172/JCI173107

**Published:** 2024-03-01

**Authors:** Orhan Efe, Rodrigo B. Gassen, Leela Morena, Yoshikazu Ganchiku, Ayman Al Jurdi, Isadora T. Lape, Pedro Ventura-Aguiar, Christian LeGuern, Joren C. Madsen, Zachary Shriver, Gregory J. Babcock, Thiago J. Borges, Leonardo V. Riella

**Affiliations:** 1 Center for Transplantation Sciences, Department of Surgery,; 2 Division of Nephrology, Department of Medicine, and; 3 Division of Cardiac Surgery, Department of Surgery, Massachusetts General Hospital, Harvard Medical School, Boston, Massachusetts, USA.; 4 Visterra Inc., Waltham, Massachusetts, USA.

**Keywords:** Immunology, Transplantation, Organ transplantation, Tolerance

## Abstract

Long-term organ transplant survival remains suboptimal, and life-long immunosuppression predisposes transplant recipients to an increased risk of infection, malignancy, and kidney toxicity. Promoting the regulatory arm of the immune system by expanding Tregs may allow immunosuppression minimization and improve long-term graft outcomes. While low-dose IL-2 treatment can expand Tregs, it has a short half-life and off-target expansion of NK and effector T cells, limiting its clinical applicability. Here, we designed a humanized mutein IL-2 with high Treg selectivity and a prolonged half-life due to the fusion of an Fc domain, which we termed mIL-2. We showed selective and sustainable Treg expansion by mIL-2 in 2 murine models of skin transplantation. This expansion led to donor-specific tolerance through robust increases in polyclonal and antigen-specific Tregs, along with enhanced Treg-suppressive function. We also showed that Treg expansion by mIL-2 could overcome the failure of calcineurin inhibitors or costimulation blockade to prolong the survival of major-mismatched skin grafts. Validating its translational potential, mIL-2 induced a selective and sustainable in vivo Treg expansion in cynomolgus monkeys and showed selectivity for human Tregs in vitro and in a humanized mouse model. This work demonstrated that mIL-2 can enhance immune regulation and promote long-term allograft survival, potentially minimizing immunosuppression.

## Abstract 2

A humanized mutein IL-2 selectively expands and boosts the function of mouse, non-human primate, and human regulatory T cells, prolonging allograft survival.

## Graphical abstract

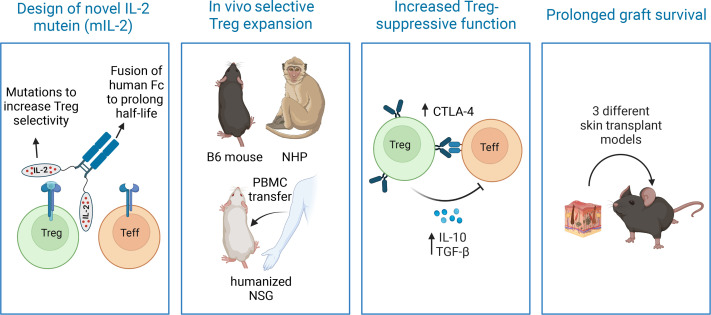

## Introduction

Immunosuppression is crucial in solid organ transplantation to prevent allograft rejection. Current approaches involve combinations of drugs targeting different immune components. These include calcineurin inhibitors (e.g., tacrolimus), antiproliferative agents (e.g., mycophenolate mofetil), and corticosteroids (1–3). Despite short-term improvements, long-term allograft survival rates remain suboptimal (3–7), with increased risks of infection, malignancy, and kidney failure (8–10). Last, the majority of currently used immunosuppressive drugs have a negative effect on immune regulation (11). Thus, alternative strategies are needed to enhance the regulatory arm of the immune system to prevent chronic allograft rejection and minimize the dose and toxicity of current immunosuppressive agents.

The expansion of Tregs is a promising strategy to reduce the risk of rejection and minimize immunosuppression. Tregs are key mediators of peripheral tolerance and modulate the effector arm of the immune system by expressing suppressive surface markers and secreting inhibitory cytokines, controlling the alloimmune response (12, 13). Depleting Tregs raises the risk of allograft rejection (14–17). IL-2 is indispensable for the function and survival of Tregs (14). Tregs constitutively express high-affinity IL-2R α subunit (IL-2Rα, also known as CD25), which renders them highly sensitive to low doses of IL-2 in contrast to effector immune cells such as CD8^+^ T cells and NK cells (14, 18). Strategies to expand Tregs in transplantation include the adoptive transfer of Tregs (19–21) or low-dose IL-2 (22–24). However, challenges remain for cellular therapy, such as defining the optimal cell source, dose, polyclonal versus antigen-specific versus engineered cells, and strategies to improve the transiency of cell survival after the adoptive transfer (25, 26). While low-dose IL-2 has been used to expand the endogenous pool of Tregs and successfully control chronic graft-versus-host disease (GVHD) and selected autoimmune diseases (27–29), similar efficacy has not been observed in organ transplantation (30, 31). Low-dose IL-2 failed to reduce immunosuppression in liver transplant recipients despite the expansion of Tregs (30). This was partly attributed to the nonselective effects of IL-2 on effector immune cells such as CD8^+^ T cells and NK cells. Another barrier to the clinical use of low-dose IL-2 is its short half-life (32), requiring multiple dosing at close intervals. Developing therapeutic approaches that selectively and sustainably expand Tregs could dramatically improve the safety and efficacy of current immunosuppression strategies.

In this study, we designed a novel IL-2 mutant protein with a prolonged plasma half-life and the ability to expand Tregs selectively (termed mIL-2). We present the molecular characteristics and pharmacokinetics of the mIL-2 and its selective and sustainable Treg expansion activity in mice. We also show that mIL-2 prolonged allograft survival and promoted antigen-specific tolerance in different murine skin transplantation models, even after the interruption of mIL-2 treatment. mIL-2 not only expanded Tregs but also enhanced their suppressive activity. Last, we demonstrate the tropism of mIL-2 for human Tregs using an in vitro phosphorylated STAT5 (p-STAT5) assay and a humanized NOD SCID γ (NSG) mouse model, and document its capacity to selectively expand Tregs in cynomolgus monkeys.

## Results

### Design of an optimal IL-2 mutein with a prolonged half-life and enhanced Treg selectivity.

Human IL-2 has pleiotropic effects on Tregs and effector immune cells such as CD8^+^ T cells and NK cells (18) and has a very short in vivo half-life of 5–7 minutes in humans and 3–4 minutes in mice (32, 33). To overcome these limitations, we first developed a mechanistic model of common γ-chain receptor cytokines by incorporating the structure of receptor-ligand interactions and molecular trafficking (34). Then, we used this model to investigate the signaling bias introduced through structural modification of IL-2, specifically modulating affinity for either the high-affinity receptor (CD25/CD122/CD132) or the intermediate receptor (CD122/CD132), or both. We aimed to identify a putative IL-2 mutein that would possess the greatest Treg specificity compared with conventional T cells and NK cells, which predominantly express the CD122/CD132 intermediate affinity receptor. From this analysis, we determined that an optimal IL-2 mutein would exhibit (a) reduced affinity for the CD122/CD132 receptor and (b) maintain, as much as possible, a high affinity for CD25. Therefore, we sought to engineer an IL-2 variant that would exhibit these attributes as much as possible.

**Figure 1:**
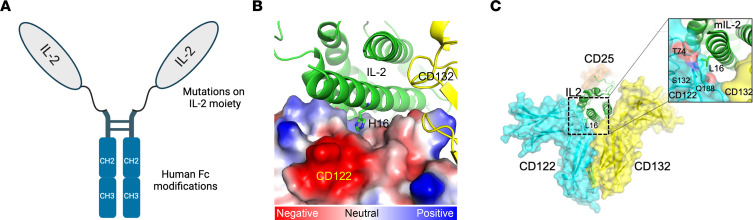
Structure of mIL-2 including its Fc domain and interactions of IL-2 with CD122 (IL-2Rβ). (**A**) Illustration of the structure of mIL-2 including the mutations to increase Treg selectivity and developability and the fusion of a modified human antibody Fc to increase its half-life. (**B**) Quaternary structure of WT IL-2 and its interaction with the IL-2R by depiction of the electrostatic charges on the surface of CD122 (IL-2Rβ). IL-2 and CD132 (IL-2Rγ) molecules are shown in green and yellow, respectively. The electrostatic structure on the surface of CD122 is depicted by a red-to-blue color gradient representing a negative-to-positive charge gradient, respectively, while the white color represents a neutral charge or a hydrophobic surface. The H16 side chain of IL-2 undergoes protonation in the endosome due to acidic pH, and the positively charged H16 is expected to enhance the electrostatic interactions between IL-2 and CD122. (**C**) Quaternary structure of mIL-2 (H16L substitution) and its interaction with the IL-2R. The IL-2 surface residue H16L substitution is expected to have minimal impact on the secondary and tertiary structures of the IL-2 molecule since leucine also has a high α helix propensity. The H16L-IL-2 molecule is shown in green, and the receptors CD25 (IL2-Ra), CD122(IL2-Rβ) and CD132(IL-2Rγ) are shown in the semitransparent surface diagram in orange, cyan and yellow, respectively. The insert shows the molecular environment of the L16 residue of mIL-2, in which the hydrophobic side chain of L16 is proximal to the polar residues of CD122 (IL-2b), and this unfavorable molecular environment is responsible for the reduced affinity of mIL-2 for CD122 at physiological conditions. The reduced affinity of mIL-2 for CD122 in physiological conditions gives a better selectivity for CD25-expressing cells, whereas in the endosome, it enables enhanced recycling.

As a baseline for additional engineering efforts and to significantly increase the half-life of IL-2 in vivo, we fused IL-2 to the Fc domain of a human IgG1 (Figure 1A) (35) with Fc modifications (see Methods) to abrogate effector functions. Binding assessments of both C-terminal and N-terminal Fc–IL-2 fusions to CD122/CD132 demonstrated that N-terminal fusions retained near-native CD25 affinity while modestly reducing CD122/CD132 affinity (Table 1), whereas C-terminal fusion had a seemingly greater effect on CD25 binding. As such, N-terminal fusion was chosen as the base molecular organization of our modified IL-2. Of note, the N-terminal fusion of IL-2 to human IgG1–Fc demonstrated modest stability issues. To increase the stability of the construct, we utilized yeast display of variants as well as a structural analysis of publicly available information to identify 3 amino acid substitutions (V69A/Q74P/C125S) within IL-2 that abrogated expression and aggregation liabilities in the fusion protein. In detailed titration studies, we found that V69A and Q74P had no effect on the binding affinity of CD25 (data not shown). Instead, these mutations seemingly cooperate to increase expression and promote an IL-2 molecule that has reduced aggregation propensity and maintains native binding to CD25.

To identify substitutions that satisfied our criteria of lowering the binding affinity to CD122/CD132 while maintaining near-WT affinity for CD25, we examined a library of muteins by yeast surface display (YSD). Error-prone PCR was used to introduce mutations on top of the V69A/Q74P/C125S background with an average of 3 mutations per clone. The mutein library was stained with varying concentrations of CD122/CD132 dimer, and a series of sorting gates were used to isolate cell populations with decreasing binding affinity. The resulting sorted libraries were analyzed using next-generation sequencing (NGS) to identify substitutions that reduced binding affinity.

Within the set of candidate substitutions, the H16 position was intriguing because it has clear structural importance for interaction with CD122. Additionally, H16 is unique among residues at the interface with CD122 because it has the potential for pH-dependent interactions with the receptor. At physiological pH, H16 forms a weak interaction with a negatively charged patch on CD122 (Figure 1B). IL-2 signaling complexes rapidly undergo endocytosis followed by either recycling to the cell surface or degradation in the lysosomes (36). At endosomal pH, H16 will become protonated, which is expected to increase binding to the negative surface of CD122. CD122 is preferentially trafficked to the lysosome, so disrupting the interaction at endosomal pH may affect the signaling of an IL-2 molecule in ways that are not explained by CD122 affinity alone. This raises the possibility that H16 substitutions have the potential to outperform previously identified substitutions in IL-2 that confer Treg selectivity, including those at position N88, which has been identified previously (37, 38). Therefore, from this effort, we identified mIL-2, a mutein that has an H16L substitution, which is predicted to have no effect on the IL-2 structure, while providing WT affinity for CD25 and significantly lower affinity for CD122/CD132 (Table 1). By replacing the polar histidine residue with a hydrophobic leucine, the side chain of position 16 now creates an unfavorable environment for interaction with the polar surface of the CD122 protein (Figure 1C), at both neutral pH and endosomal pH.

### Prolonged half-life and in vitro Treg selectivity of mIL-2.

**Figure 2:**
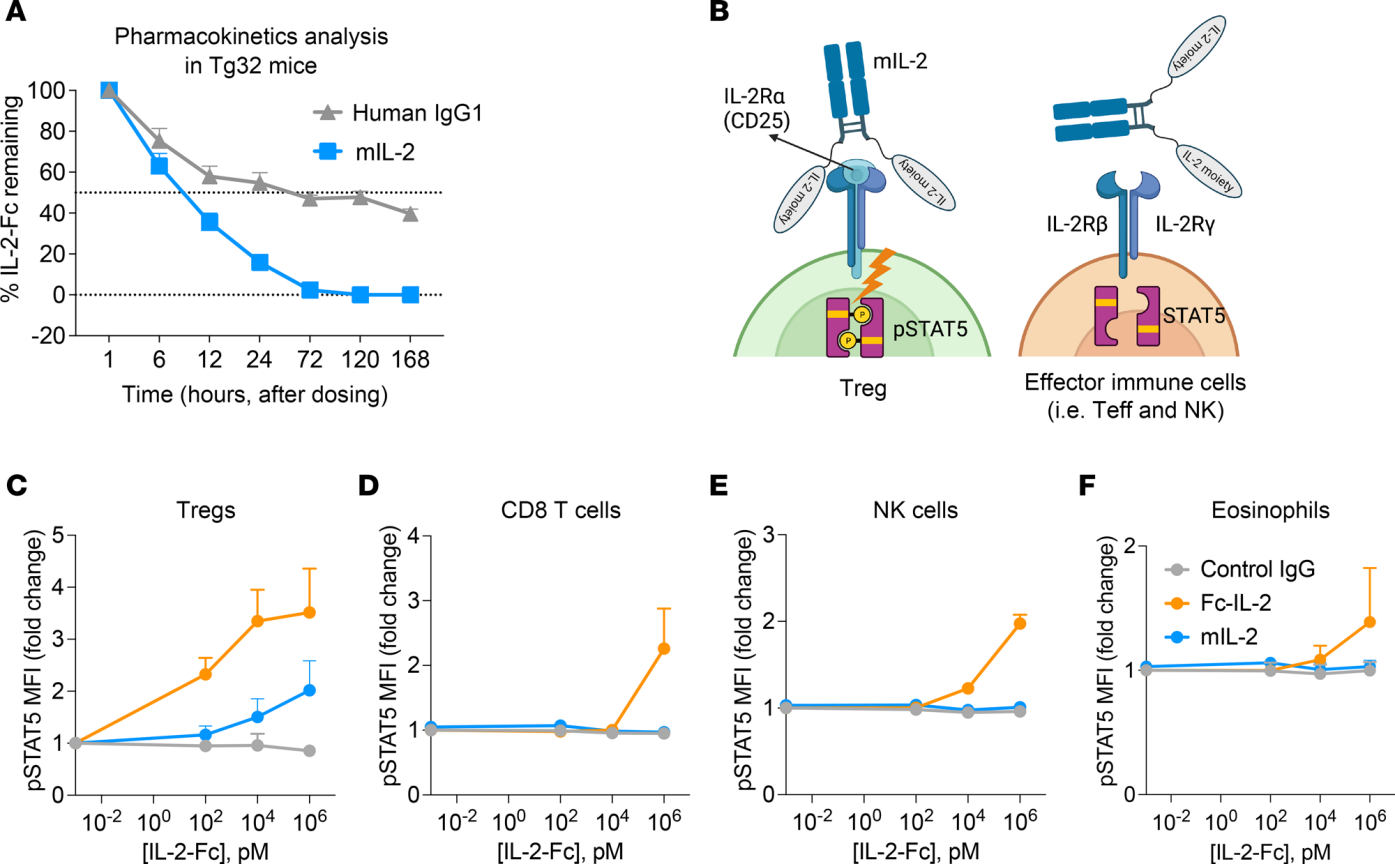
The Treg specificity and pharmacokinetics of mIL-2. (**A**) Bioavailability of intravenous mIL-2 (0.25 mg/kg) compared with equimolar human IgG1 (1.5 mg/kg) in hFcRn-transgenic Tg32 mice, indicating its prolonged half-life (*n* = 3/group, data are from a single experiment). (**B**) Illustration showing the selective binding of mIL-2 to Tregs but not to effector immune cells due to constitutive IL-2Rα (CD25) expression on Tregs. Flow cytometric analyses of p-STAT5 (pY694) levels in splenic (**C**) Tregs, (**D**) CD8^+^ T cells, (**E**) NK cells, and (**F**) eosinophils after a 30-minute in vitro stimulation with control IgG, mIL-2, or Fc–IL-2 at increasing concentrations (*n* = 5/group, data were pooled from 3 independent experiments). Graphs show the mean ± SD and 1-way ANOVA and Tukey’s multiple-comparison test were used for group comparisons (**C**–**F**).

To test the molecular characteristics of mIL-2, we first performed a pharmacokinetics analysis. Following intravenous injection of mIL-2 or equimolar human IgG1 into Tg32 mice that carried a human neonatal Fc receptor (FcRn) for human IgG recycling (39), we measured the circulating levels of mIL-2 and IgG1 by ELISA (*n* = 3/group). mIL-2 had a half-life of 6–12 hours, which is remarkably longer than recombinant human IL-2 (32), and remained in circulation for up to 72 hours. A representative human IgG1 had a half-life of 24–72 hours in this experiment (Figure 2A).

To test the Treg selectivity of mIL-2 (Figure 2B), we stimulated mouse splenocytes in vitro with increasing concentrations of mIL-2, control IgG, or WT Fc–IL-2 (referred to hereafter as Fc–IL-2) for 30 minutes and measured the levels of p-STAT5 (pY694), which is a downstream effector of the IL-2 receptor (14), in Tregs and effector immune cells. As expected, Fc–IL-2 increased p-STAT5 MFI nonselectively in Tregs (Figure 2C), CD8 T^+^ cells (Figure 2D), NK cells (Figure 2E), and eosinophils (Figure 2F). In contrast, mIL-2 increased p-STAT5 MFI only in Tregs in a dose-dependent manner (Figure 2C) and did not affect p-STAT5 levels in CD8^+^ T cells (Figure 2D), NK cells (Figure 2E), or eosinophils (Figure 2F) over a wide dose range compared with controls. In sum, these findings show the prolonged in vivo half-life and enhanced Treg selectivity of mIL-2 in vitro.

### Selective and sustainable in vivo Treg expansion by mIL-2.

To test in vivo Treg expansion by mIL-2, we first treated naive C57BL/6 (B6) mice with a single subcutaneous or intravenous injection of mIL-2 at 0.5 mg/kg or a subcutaneous injection of vehicle (PBS). Both subcutaneous and intravenous injections of mIL-2 led to an approximately 4.5-fold expansion of circulating Tregs by day 3 compared with controls, with an effect that lasted for 7 days (Supplemental Figure 1A; supplemental material available online with this article; https://doi.org/10.1172/JCI173107DS1). Circulating CD8^+^ T cells (Supplemental Figure 1B), NK cells (Supplemental Figure 1C), and eosinophils (Supplemental Figure 1D) remained similar between the groups. Since intravenous and subcutaneous injections of mIL-2 showed similar effects, we used the subcutaneous route for the subsequent experiments, as it is more convenient for clinical use.

**Figure 3:**
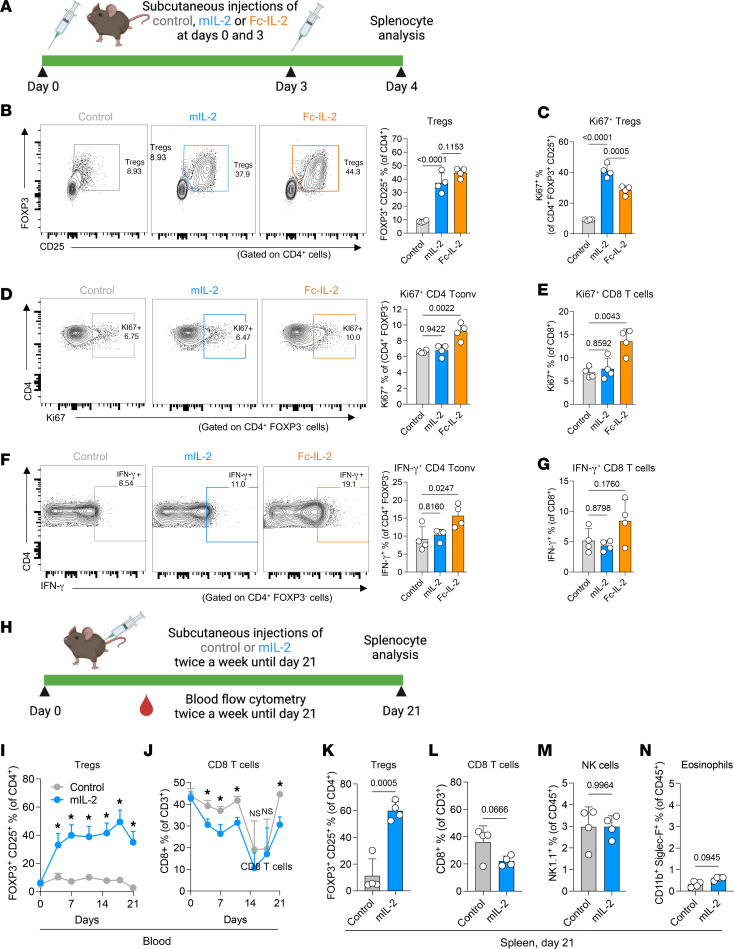
In vivo selective Treg expansion by mIL-2. (**A**) Illustration of the short-term in vivo experiment in which B6 FOXP3-GFP mice received 2 doses of control IgG, mIL-2, or Fc–IL-2 on days 0 and 3, and spleens were harvested on day 4. (**B**) Representative flow cytometry plot and bar graph showing the frequencies of splenic total Tregs. (**C**) Bar graph showing the frequencies of Ki67^+^ Tregs. (**D**) Representative flow cytometry and bar graph showing the frequencies of Ki67^+^CD4^+^ Tconv cells. (**E**) Bar graph showing the frequencies of Ki67^+^CD8^+^ T cells. (**F**) Representative flow cytometry and bar graph showing the frequencies of IFN-γ^+^CD4^+^ Tconv cells. (**G**) Bar graph showing the frequencies of IFN-γ^+^CD8^+^ T cells following in vitro stimulation with a PMA/ionomycin/brefeldin cocktail for 6 hours (*n* = 4/group; data are representative of 2 independent experiments). (**H**) Illustration showing the sustained treatment experiment in which B6 FOXP3-GFP mice received twice-weekly subcutaneous injections of mIL-2 or control IgG for 21 days. Flow cytometric analyses of peripheral (**I**) Tregs and (**J**) CD8^+^ T cells over time, and splenic (**K**) Tregs, (**L**) CD8^+^ T cells, (**M**) NK cells, and (**N**) eosinophils on day 21 (*n* = 4/group, data representative of single experiment). In all experiments, mIL-2 and Fc–IL-2 were given at 0.5 mg/kg, and control IgG was given at an equimolar dose. Graphs show the mean ± SD. One-way ANOVA with Tukey’s multiple-comparison test was used for 3-group comparisons (**B**–**G**) and unpaired 2-tailed *t* test for 2-group comparisons (**I**–**N**) (*P ≤ 0.05; NS, *P* > 0.05).

We then investigated splenic Treg expansion following 2 subcutaneous injections of mIL-2, Fc–IL-2, or equimolar control IgG into B6 mice (Figure 3A). Compared with controls, both mIL-2 and Fc–IL-2 treatments led to a 4- to 5-fold expansion of Tregs at day 4 (Figure 3B). Both also increased the percentages of proliferating (Ki67^+^) Tregs (Figure 3C). With regard to the expansion of effector T cells, only Fc–IL-2 treatments increased the proportion of Ki67^+^ (Figure 3, D and E) CD4^+^ conventional T (Tconv) cells and CD8^+^ T cells compared with controls. Moreover, Fc–IL-2, but not mIL-2, increased the percentage of IFN-γ^+^ CD4^+^ Tconv and CD8^+^ T cells (Figure 3, F and G) compared with controls; however, the latter did not reach statistical significance (*P* = 0.176). These findings highlight the selectivity of mIL-2 compared with that of Fc–IL-2.

The lack of sustainability of Treg expansion is one of the limitations of Treg-centric strategies such as adoptive transfer of Tregs (19, 40). To test the sustainability of Treg expansion by mIL-2, we treated B6 mice with mIL-2 or control IgG twice per week for 21 days (Figure 3H). mIL-2 injections led to a stable expansion of circulating Tregs starting after the first dose until day 21 (Figure 3I). This was accompanied by a decrease in circulating CD8^+^ T cells by approximately 25% (Figure 3J). Circulating NK cells and eosinophils were similar between the mIL-2 and control groups (data not shown). Similar to blood, mIL-2 treatment expanded Tregs (Figure 3K) and reduced CD8^+^ T cells (Figure 3L) compared with controls in the spleen, although the latter did not reach statistical significance (*P* = 0.0666). Splenic NK cells (Figure 3M) and eosinophils (Figure 3N) were similar in the mIL-2 and control groups. Thus, these findings show the durability and selectivity of in vivo Treg expansion by mIL-2 both in the circulation and secondary lymphoid organs.

### Efficacy of mIL-2 in minor-mismatch skin transplant models.

**Figure 4:**
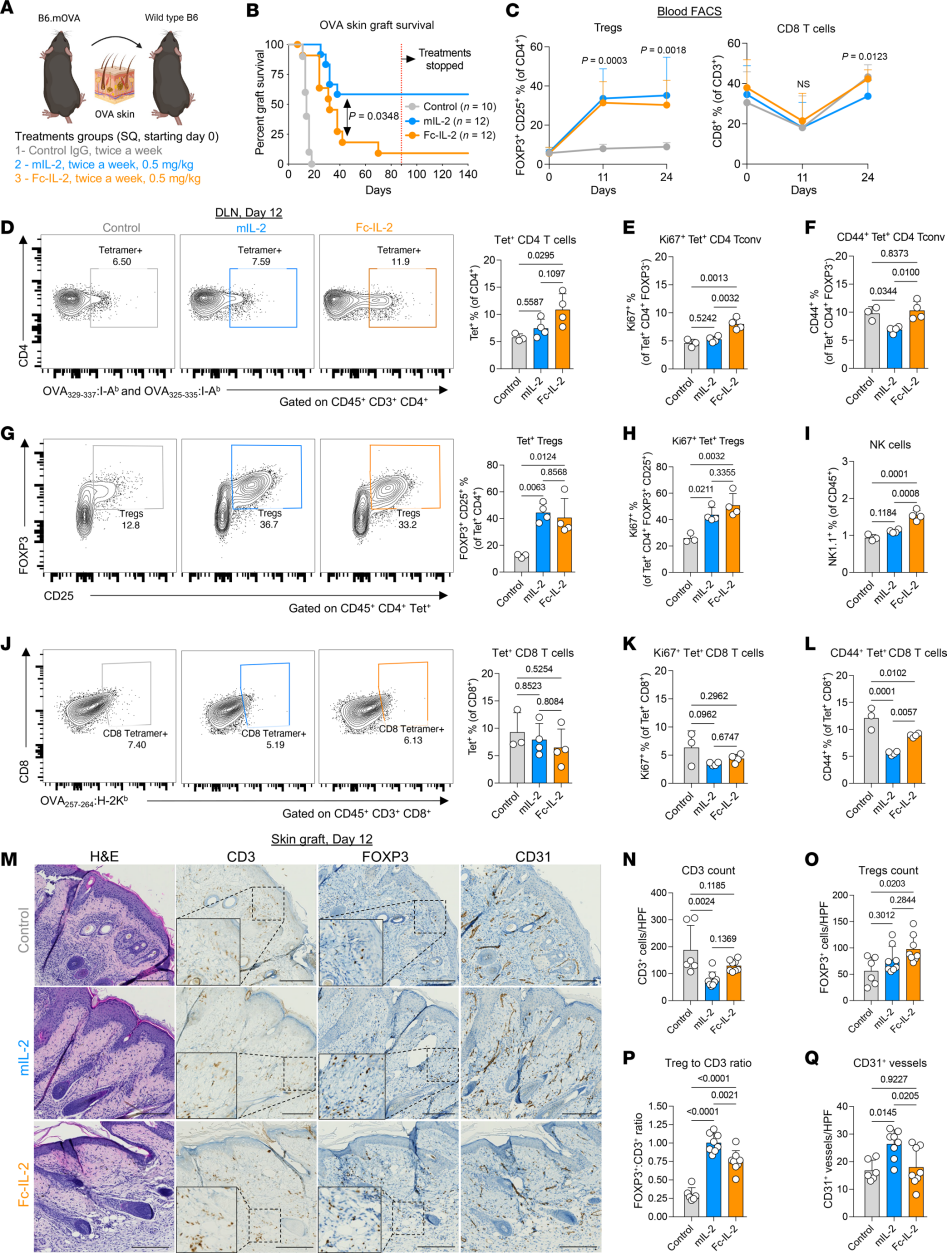
Effect of mIL-2 in a minor-mismatch skin transplant model. (**A**) Illustration of the experimental design and (**B**) Kaplan-Meier graph of the graft survival in B6.mOVA to WT B6 mouse skin transplantation (*n* = 10–12/group, data are representative of 3 independent experiments). (**C**) Flow cytometric analyses of peripheral Tregs and CD8^+^ T cells on days 0, 11, and 24. (**D**) Representative flow cytometric gating and analysis of OVA tetramer^+^CD4^+^ T cells using a combination of OVA_329–337_:I-A^b^ and OVA_325–335_:I-A^b^ tetramers and graph analyses showing the percentages of (**E**) Ki67^+^ and (**F**) CD44^+^tetramer^+^CD4^+^ Tconv cells in the DLNs on day 12. (**G**) Representative flow cytometric gating and analysis of the total tetramer^+^ Tregs and (**H**) percentages of Ki67^+^tetramer^+^ Tregs. (**I**) Analysis of the frequencies of NK cells. (**J**) Representative flow cytometry gating and percentages CD8^+^ T cells positive for the OVA_257–264_:H-2K^b^ tetramer and frequencies of (**K**) Ki67^+^ and (**L**) CD44^+^ cells in CD8^+^tetramer^+^ T cells (*n* = 4/group). (**M**) Representative images of H&E staining and IHC with antibodies against FOXP3 and CD3 of the skin grafts at day 12. Dashed line–outlined rectangular areas are shown at ×2 higher magnification on the left lower corners of the FOXP3 and CD3 IHC images. Graphs show the counts of (**N**) CD3^+^ and (**O**) FOXP3^+^ cells, (**P**) the ratios of FOXP3^+^ to CD3^+^ cell counts, and (**Q**) the counts of CD31^+^ vessels in 2 random high-powered fields per graft (*n* = 4/group, data are representative of a single experiment). Scale bars: 50 μm. Graphs show the mean ± SD and 1-way (**D**–**Q**) or 2-way (**C**) ANOVA with Tukey’s multiple-comparison test was used for the group comparisons. The long-rank test was used for graft survival comparisons (**B**). HPF, high-powered field.

To test the therapeutic efficacy of mIL-2 in transplantation, we first used 2 minor-mismatch skin transplant models: OVA-expressing B6 (B6.mOVA) donors to WT B6 recipients and B6 male donors to B6 female recipients. B6.mOVA mice express cell-surface OVA on cells of all organs and are used as donors in this minor histocompatibility antigen transplant model. In the OVA skin transplant model, the recipient mice were treated with mIL-2, Fc–IL-2, or control IgG subcutaneously twice a week, starting on day 0 and continued until week 12 after transplantation (Figure 4A). mIL-2 treatment led to prolonged graft survival compared with both the Fc–IL-2 and control groups, with a median transplant survival (MTS) of 151.5, 32, and 14 days, respectively (*P* < 0.0001) (Figure 4B). Interestingly, the grafts survived long term (>140 days), even after treatments were discontinued at week 12. Mechanistically, both mIL-2 and Fc–IL-2 treatments expanded circulating Tregs by approximately 4-fold after transplantation (Figure 4C). In contrast to Fc–IL-2, mIL-2 decreased the percentages of CD8^+^ T cells compared with the control groups at day 24 (Figure 4C). Circulating NK cell percentages were similar in all groups (Supplemental Figure 2A). Compared with controls, circulating eosinophils were increased by Fc–IL-2 (*P* = 0.0054), but not mIL-2, at day 24 after transplantation (*P* = 0.24) (Supplemental Figure 2B).

Tetramers are molecular tools composed of a specific peptide bound to an MHC molecule and are used to track antigen-specific T cells (41). By using OVA: I-A^b^ tetramers, we next tracked OVA donor–specific (Tet^+^) CD4^+^ T cells in the draining lymph nodes (DLNs) of the skin grafts at day 12 after transplantation. The the mean percentage of Tet^+^CD4^+^ T cells was increased by Fc–IL-2 but was unchanged in the mIL-2 group compared with controls (Figure 4D). Among the Tet^+^CD4^+^ Tconv (FOXP3^–^) cells, mIL-2 did not change the percentage of Ki67^+^ cells, while the percentage was increased by Fc–IL-2 compared with controls (Figure 4E). The mIL-2 treatment decreased the percentage of CD44^+^ (Figure 4F) and CD69^+^Tet^+^CD4^+^ Tconv (Supplemental Figure 2C) cells compared with both the control and Fc–IL-2 groups. Regarding antigen-specific Tregs, both mIL-2 and Fc–IL-2 increased Tet^+^ Tregs (CD4^+^FOXP3^+^) compared with controls (Figure 4G), as well as the percentages of proliferating Ki67^+^Tet^+^ Tregs (Figure 4H). NK cells were increased by Fc–IL-2 but not by mIL-2 (Figure 4I). The frequencies of Tet^+^ CD8^+^ T cells were similar between the groups (Figure 4J), but mIL-2 decreased the percentages of Ki67^+^ (Figure 4K) compared with controls, although this was not statistically significant (*P* = 0.0962). mIL-2 also decreased CD44^+^ (Figure 4L) and CD69^+^Tet^+^CD8^+^ T cells (Supplemental Figure 2D) compared with both the control and Fc–IL-2 groups (Supplemental Figure 2D). Last, eosinophils were increased by mIL-2 (*P* = 0.0018) and by Fc–IL-2 (*P* = 0.0942) compared with controls (Supplemental Figure 2E).

Consistent with the graft survivals and mechanistic experiments of the DLNs, the immunohistochemical analysis of the skin grafts showed fewer CD3^+^ cells and more graft-infiltrating FOXP3^+^ cells in mIL-2–treated mice compared with controls (Figure 4, M–O). Strikingly, almost all the intra-graft CD3^+^ cells were Tregs in the mIL-2 group, as reflected by the fact that the ratio of FOXP3^+^ to CD3^+^ cell counts was close to 100% versus 30% in the controls (Figure 4P). Although Fc–IL-2 also increased the FOXP3^+^ cell count, the ratio of FOXP3^+^ to CD3^+^ cell counts was lower than that in the mIL-2 group (75%) (Figure 4P), suggesting a superior ability of mIL-2 to induce tissue-resident Tregs. We also performed CD31 IHC staining to label blood vessels in the graft, since vascularization is a marker of skin graft survival (42). Correlating with the graft survivals, we found that CD31^+^ vessel counts were higher in the mIL-2 group compared with both the control and Fc–IL-2 groups (Figure 4Q).

Last, we checked plasma donor–specific anti-OVA antibodies at baseline and on days 12 and 21 after transplantation to assess the humoral response to the graft. In the mIL-2 group, none of the mice developed anti-OVA antibodies compared with controls and Fc–IL-2–treated mice (Supplemental Figure 2F). These data suggest that mIL-2 treatment expanded polyclonal and donor-specific Tregs and was associated with the inhibition of donor-specific antibodies, prolonging allograft survival.

To validate our findings, we tested the efficacy of mIL-2 in a male-to-female B6 skin transplant model (Supplemental Figure 3A). The recipient mice were treated with mIL-2 or vehicle (PBS) twice a week, starting on day 0. mIL-2 treatment prolonged the MTS compared with controls (>212 days vs. 34.5 days, *P* = 0.0009) (Supplemental Figure 3B). Similar to the OVA skin transplant model, the grafts from the mIL-2 group survived long term, even after treatments were stopped at 13 weeks after transplantation. mIL-2 treatment led to an expansion of circulating Tregs (Supplemental Figure 3C) and decreased CD8^+^ T cell percentages, which were sustained until day 21 (Supplemental Figure 3D). Circulating NK cells (Supplemental Figure 3E) and eosinophils (Supplemental Figure 3F) remained similar in both groups. We also checked the long-term sustainability of circulating Treg expansion. Immediately prior to the discontinuation of the mIL-2 treatments at week 13, Treg percentages were still expanded at a mean of 38% of the pool of CD4^+^ T cells, and this expansion started to wane within 7–21 days after discontinuation of mIL-2 (Supplemental Figure 4). In sum, mIL-2 could prolong allograft survival in 2 minor-mismatched models, even after treatment discontinuation with selective Treg expansion.

### Effect of mIL-2 in a major-mismatch skin transplant model.

**Figure 5:**
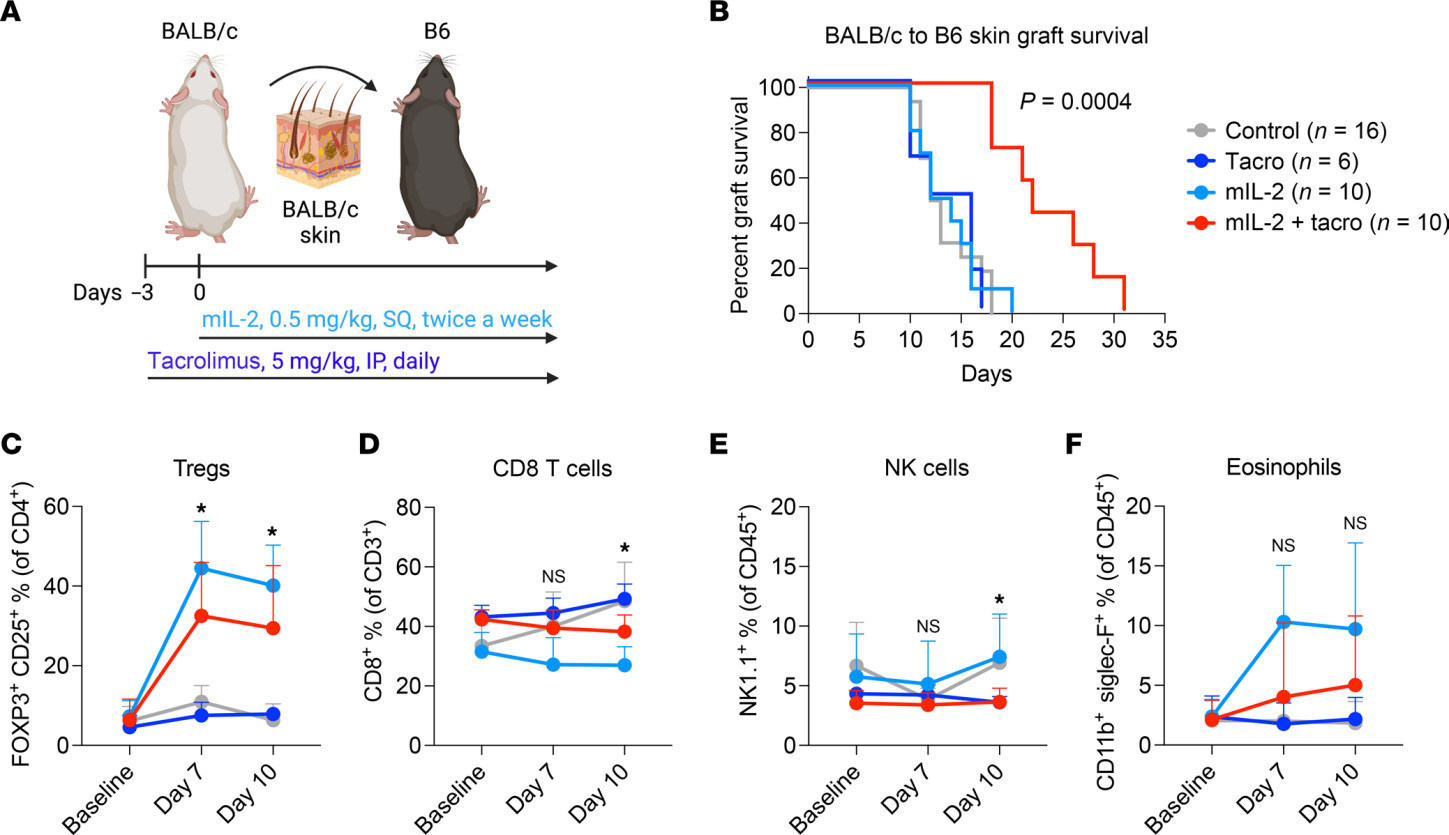
Effect of mIL-2 in combination with tacrolimus in a major-mismatch skin transplant model. (**A**) Illustration of the BALB/c to B6 skin transplantation model, in which tacrolimus was given daily intraperitoneally at 5 mg/kg starting at day -3 and mIL-2 was given subcutaneously twice a week at 0.5 mg/kg starting on day 0. (**B**) Representative survival curve of the skin allografts in different treatment groups (*n* = 6–16, data were pooled from 5 independent experiments). Graphs of flow cytometric analyses of circulating (**C**) Tregs, (**D**) CD8^+^ T cells, (**E**) NK cells, and (**F**) eosinophils at baseline and on days 7 and 10. Graphs show the mean ± SD, and 2-way ANOVA with Tukey’s multiple-comparison test was used for group comparisons (**C**–**F**). The long-rank test was used for graft survival comparisons (**B**). *P ≤ 0.05; NS, *P* > 0.05, comparing the mIL-2 plus tacrolimus group versus the control group.

To test mIL-2 in a major-mismatch skin transplant model, we used BALB/c to B6 skin transplantation, in which graft rejection occurs within 10–12 days without treatments (43). It is considered one of the most stringent transplant models compared with other organ transplants such as kidney or liver (44). We treated the recipient mice with mIL-2 and/or tacrolimus (Figure 5A). The latter is commonly used as part of the immunosuppressive regimen of human transplant recipients. Neither mIL-2 nor tacrolimus alone was able to prolong the survival of the skin graft, but their combination was successful in prolonging graft survival (*P* = 0.0002) (Figure 5B). The Fc portion of the IgG molecules have been described to promote immune quiescence that potentially synergizes with calcineurin inhibitors (45, 46). To control for this effect, we treated skin transplant recipients with tacrolimus plus a control human IgG, which failed to prolong skin graft survival compared with vehicle control (Supplemental Figure 5). Circulating Tregs were significantly expanded in both the mIL-2 and mIL-2 plus tacrolimus treatment groups at days 7 and 10 after transplantation compared with controls (Figure 5C). Both mIL-2 and mIL-2 plus tacrolimus treatments decreased the percentages of circulating CD8^+^ T cells at day 10 after transplantation (Figure 5D). NK cells were decreased (Figure 5E), and eosinophils tended to increase by mIL-2 and tacrolimus treatment compared with controls at day 10 (Figure 5F). In sum, mIL-2 and tacrolimus synergized to prolong allograft survival in a major-mismatch skin transplant model.

We also tested the combination of mIL-2 with CTLA-4 Ig, a costimulation blockade therapy also used in solid organ transplant recipients, usually in place of tacrolimus. In a previous study, we demonstrated that CTLA-4 Ig could negatively affect Treg homeostasis and decrease Treg ratios (47). In BALB/c skin transplant recipient mice, CTLA-4 Ig alone failed to prolong the graft survival, but the combination of mIL-2 and CTLA-4 Ig prolonged allograft survival (*P* = 0.0191) (Supplemental Figure 6A). Circulating Tregs tended to decrease in the CTLA-4 Ig group compared with controls on days 7 and 10 (Supplemental Figure 6, B and C). On the other hand, circulating Tregs were increased by both mIL-2 alone and mIL-2 plus CTLA-4 Ig treatments (Supplemental Figure 6B). Overall, the combination of relatively low-dose immunosuppression with mIL-2 prolonged graft survival in the most stringent transplantation model.

### mIL-2 induces antigen-specific tolerance.

**Figure 6:**
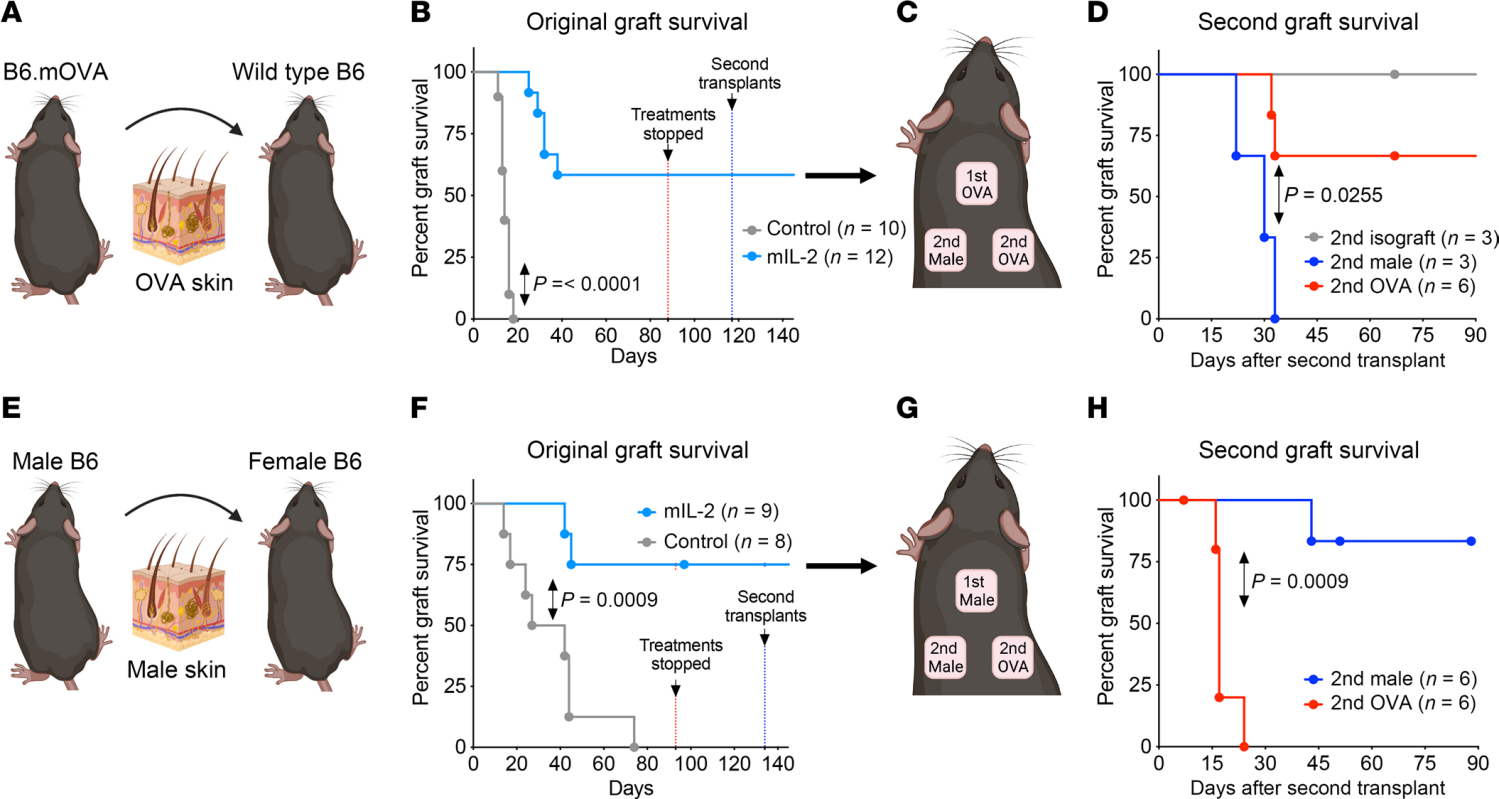
Assessment of antigen-specific tolerance after mIL-2 treatment and rechallenge with a second graft in 2 minor-mismatch skin transplant models. (**A**) Illustration of a female B6.mOVA to female WT B6 skin transplant model. (**B**) Kaplan-Meier graph showing the OVA graft survival in mIL-2 versus control groups and the days of mIL-2 treatment discontinuation (red dotted line) and the second skin transplantation (blue dotted line), respectively. (**C**) Illustration of the antigen-specific tolerance experiment, in which original OVA graft recipient mice were challenged with 2 additional grafts, including a similar graft and a third-party graft. (**D**) Kaplan-Meier graph showing the long-term survival of a second OVA graft and early rejection of a third-party (male) graft in the original OVA graft female recipients. Isograft, syngeneic graft. (**E**) Illustration of the experiment and (**F**) Kaplan-Meier graph showing the original graft survival, treatment discontinuation, and the timing of the second skin transplant in male to female B6 skin transplantation. (**G**) Illustration of the tolerance experiment in the original male graft recipients and (**H**) Kaplan-Meier graph showing the long-term survival of the second similar grafts (male graft) and early rejection of the third-party grafts (OVA graft). The long-rank test was used for graft survival comparisons (**B**, **D**, **F**, and **H**) (*n* = 3–12 animals/group, data were pooled from 5 independent experiments).

In minor-mismatch skin transplant models (B6.mOVA to WT B6 and B6 male to female) (Figure 4 and Supplemental Figure 3), skin grafts continued to survive long term (>140 days), even after discontinuation of mIL-2, which raised the question of whether these mice had developed an antigen-specific peripheral tolerance. To test this hypothesis, we challenged the original recipients with additional donor-specific grafts, third-party grafts, or isografts after the discontinuation of mIL-2 treatments and the return of circulating Treg levels to the baseline (Figure 6). In the original female OVA graft recipients, the second female OVA grafts had a prolonged survival but not the second male grafts (MTS >97 days vs. 30 days, respectively, *P* = 0.0112) (Figure 6, A–D). We observed the same effect when the second B6 male or female OVA skin grafts were transplanted onto B6 female recipients of male grafts (MTS >51 days vs. 17 days, respectively, *P* = 0.009) (Figure 6, E–H). In sum, these findings indicate the emergence of antigen-specific tolerance with mIL-2 treatment that was long lasting even after treatment interruption and the return of peripheral Treg numbers to untreated levels.

### Effects of mIL-2 on Treg-suppressive functions and the role of CTLA-4 in the efficacy of mIL-2 in skin transplantation.

**Figure 7:**
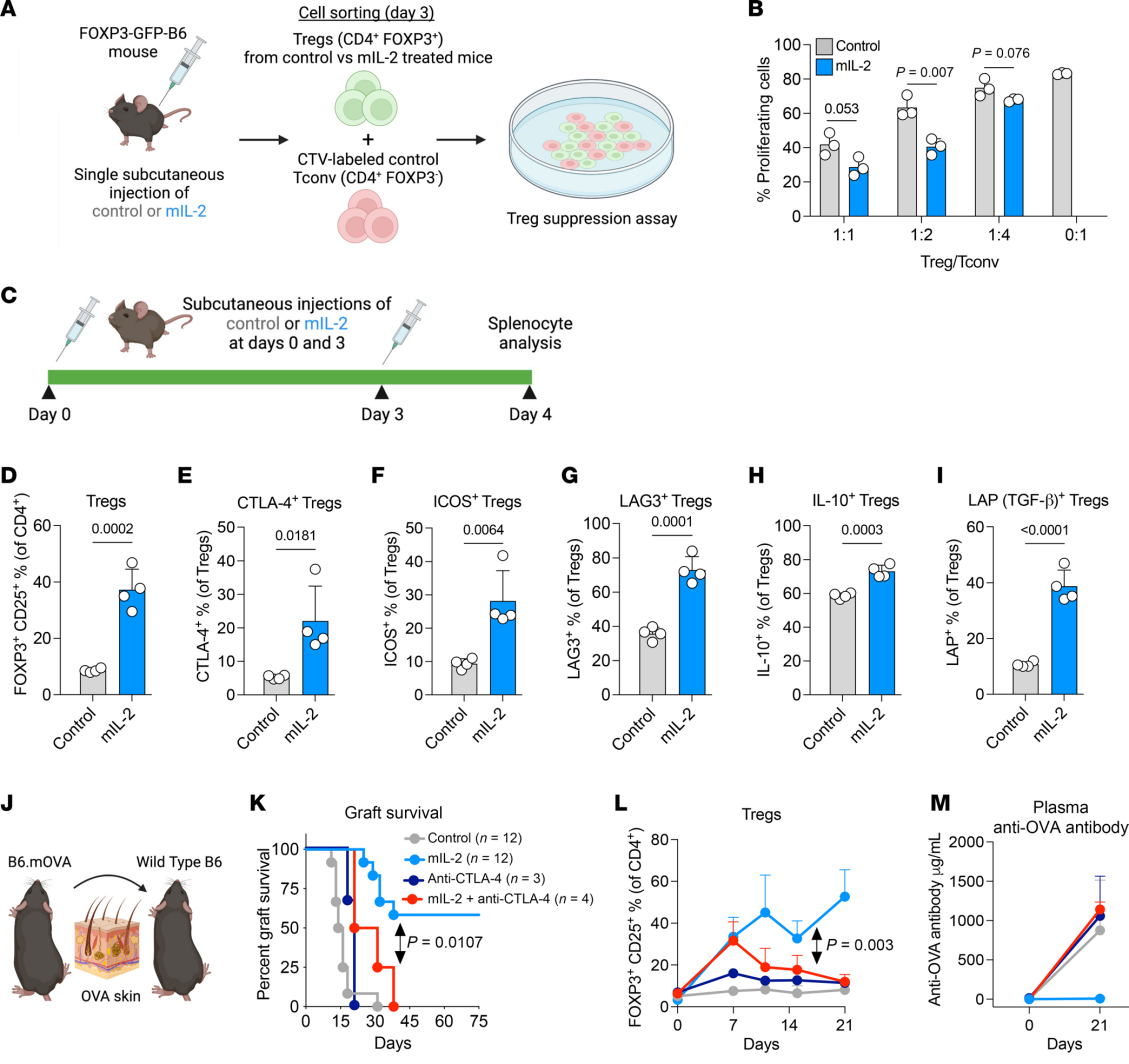
Effect of mIL-2 on Treg-suppressive function and its CTLA-4–dependent efficacy in skin transplantation. (**A**) Illustration of the Treg suppression assay performed by coculturing naive CTV-labeled CD4^+^ Tconv cells with Tregs from B6 FOXP3-GFP mice treated with control IgG or mIL-2 (0.5 mg/kg). (**B**) Percentages of proliferating CD4^+^ Tconv cells after coculturing with different ratios of Tregs (*n* = 3/group; representative data are from 3 independent experiments). (**C**) Illustration of the experimental design to assess the functional surface markers and inhibitory cytokines of splenic Tregs in which B6 FOXP3-GFP mice were treated with control IgG or mIL-2 at 0.5 mg/kg subcutaneously on days 0 and 3, and splenocytes were harvested on day 4. Flow cytometric analyses of (**D**) total Tregs, (**E**) CTLA-4^+^, (**F**) ICOS^+^, and (**G**) LAG3^+^ Tregs. Flow cytometric analyses of (**H**) IL-10^+^ and (**I**) LAP^+^ (TGF-β^+^) Tregs after in vitro stimulation with a PMA/ionomycin/brefeldin cocktail for 6 hours (*n* = 4/group, data are representative of 2 independent experiments). (**J**) Illustration of B6.mOVA to WT B6 skin transplantation, in which the recipient mice were treated with subcutaneous control IgG or mIL-2 (0.5 mg/kg) with or without anti–CTLA-4 antibodies (combination of 2 antibodies from the clones of UC10-4F10-11 and 9H10; 200 μg/mouse for each clone, intraperitoneally) twice a week starting on day 0 (*n* = 3–12/group, data were pooled from 4 different experiments). (**K**) Kaplan-Meier curves of the graft survivals. (**L**) Flow cytometric analysis of peripheral blood Tregs from the recipient mice. (**M**) Plasma anti-OVA antibody levels as determined by ELISA on days 0 and 21 (*n* = 3–4/group). Graphs show the mean ± SD. A 2-tailed *t* test was used for 2-group comparisons (**B** and **D**–**I**) and 1-way (**M**) and 2-way (**L**) ANOVA with Tukey’s multiple-comparison test for 3 or more group comparisons as appropriate. The long-rank test was used for graft survival comparisons (**K**). CTV, CellTrace Violet.

IL-2 promotes the function of Tregs through increased suppressive surface proteins and inhibitory cytokine secretion, which might play a role in the prolongation of skin graft survival (12). To investigate the effects of mIL-2 on Treg-suppressive abilities, we performed an in vitro suppression assay in which naive CD4^+^ Tconv cells were cocultured with Tregs sorted from mIL-2 versus control IgG–treated B6 FOXP3-GFP mice (Figure 7A). Tregs from mIL-2–treated mice exhibited a greater ability to suppress CD4^+^ Tconv cell proliferation when they were cocultured at different ratios (Figure 7B). To delineate the mechanisms of increased Treg functions by mIL-2, we measured Treg functional surface markers and inhibitory cytokine secretion in the splenocytes of B6 mice following 2 subcutaneous injections of mIL-2 or control IgG (Figure 7C). mIL-2 treatment expanded splenic Tregs (Figure 7D) and increased the percentages of CTLA-4^+^ (Figure 7E), ICOS^+^ (Figure 7F), and LAG-3^+^ (Figure 7G) Tregs compared with controls (Figure 7, D–G). Tregs from mIL-2–treated mice had increased production of IL-10 (Figure 7H) and LAP, a TGF-β–associated protein (Figure 7I). Since CTLA-4^+^ Tregs are essential for allogenic tolerance (48, 49) and immune modulation (50), we investigated the role of CTLA-4 in the mIL-2–induced prolongation of transplant survival. We used a combination of 2 non-Treg-depleting anti–CTLA-4 monoclonal antibodies (clone UC10-4F10-11 (49, 51, 52) and 9H10 (53)) in OVA skin graft recipient mice treated with mIL-2 or control (Figure 7J). The combination of anti–CTLA-4 with mIL-2 led to the shortening of graft survival compared with mIL-2 alone, indicating that the prolongation of allograft survival by mIL-2 depended on CTLA-4 (Figure 7K). Interestingly, circulating Treg expansion gradually weaned in the mIL-2 plus anti–CTLA-4 group from days 7–21, indicating that Treg expansion might also depend on CTLA-4 signaling (Figure 7L). Last, combining anti–CTLA-4 antibody with mIL-2 led to increased plasma donor–specific anti-OVA antibody levels compared with mIL-2 alone (Figure 7M). Taken together, these data show that mIL-2 enhanced the function of Tregs through increased functional surface markers and inhibitory cytokine secretion and that its efficacy in skin transplantation was dependent on CTLA-4 signaling.

### Effect of mL-2 in human and nonhuman primate Tregs.

**Figure 8:**
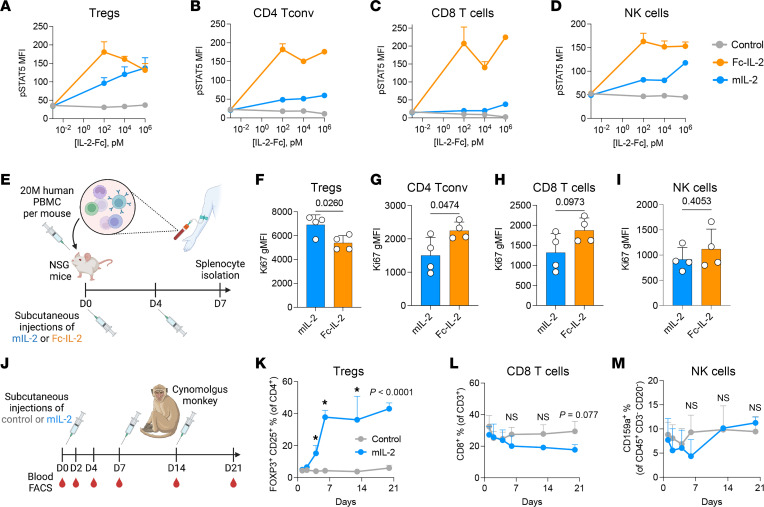
Effect of mIL-2 in human and nonhuman primate Tregs. Flow cytometric analyses of p-STAT5 levels in human PBMCs including (**A**) Tregs, (**B**) CD4^+^ Tconv cells, (**C**) CD8^+^ T cells, and (**D**) NK cells after 30-minute in vitro treatments with increasing concentrations of control IgG, mIL-2, or WT Fc–IL-2 (*n* = 3/group). (**E**) Illustration of the humanized NSG mouse model, in which 20 million human PBMCs were intravenously injected into NSG mice that were treated with subcutaneous injections of mIL-2 or Fc–IL-2 (0.05 mg/kg) on days 0 and 4 followed by spleen isolation on day 7. Flow cytometric analyses of Ki67 levels in human (**F**) Tregs, (**G**) CD4^+^ Tconv cells, (**H**) CD8^+^ T cells, and (**I**) NK cells in a humanized NSG mouse model (*n* = 4/group, data are from 2 experiments). (**J**) Illustration of the experimental design of in vivo Treg expansion in cynomolgus monkeys by subcutaneous injection of vehicle versus mIL-2 at 0.5 mg/kg on days 0, 7, and 14 and flow cytometric analysis of peripheral blood (**K**) Tregs, (**L**) CD8^+^ T cells, and (**M**) NK cells over time (*n* = 4/group). Graphs show the mean ± SD. A 2-tailed *t* test was used for 2-group comparisons (**F**–**I** and **K**–**M**), and 1-way ANOVA with Tukey’s multiple-comparison test (**A**–**D**) was used for 3-group comparisons (*P ≤ 0.05; NS, *P* > 0.05). gMFI, geometric MFI.

We designed mIL-2 as a fusion of 2 human IL-2 moieties with the Fc portion of human IgG1 (with the removal of the N-linked glycosylation at N297 to limit effector functions), with the aim of clinical use. To translate our findings from mice to humans, we first treated healthy human PBMCs in vitro with increasing concentrations of mIL-2, control IgG, or Fc–IL-2 for 30 minutes. Using flow cytometry, we evaluated p-STAT5 (Y694) levels in Tregs, CD4^+^ Tconv cells, CD8^+^ T cells, and NK cells. Both mIL-2 and Fc–IL-2 increased p-STAT5 expression in human Tregs in a dose-dependent manner compared with controls (Figure 8A). However, mIL-2 had minimal effect on p-STAT5 levels in CD4^+^ Tconv and CD8^+^ T cells, whereas Fc–IL-2 exhibited nonselective, dose-dependent effects (Figure 8, B and C). In NK cells, mIL-2 treatment had no effect on the levels of p-STAT5 except at high concentrations compared with the Fc–IL-2 group, which had increased levels starting at low concentrations (Figure 8D).

To test the in vivo effects of mIL-2 on human PBMCs, we used a humanized NSG mouse model. We transferred approximately 20 million human PBMCs per mouse and treated them with 0.05 mg/kg mIL-2 or Fc–IL-2 on days 0 and 4 and isolated the splenocytes on day 7 (Figure 8E). In comparison with Fc–IL-2, the mIL-2 group had higher percentages of Ki67^+^ Tregs (CD4^+^FOXP3^+^CD25^+^CD127^–^, Figure 8F) but lower Ki67^+^CD4^+^ Tconv cells (Figure 8G), CD8^+^ T cells (Figure 8H), and NK cells (Figure 8I), although the difference for the CD8^+^ T cells and NK cells did not reach statistical significance. These findings suggest a higher selectivity of mIL-2 than Fc–IL-2. Last, we used a nonhuman primate model to investigate in vivo Treg expansion by mIL-2 in an environment that was more similar to that in humans. We injected cynomolgus monkeys subcutaneously with mIL-2 at 0.5 mg/kg or with vehicle on days 0, 7, and 14 and performed blood flow cytometric analyses periodically until day 21 (Figure 8J). mIL-2 treatments expanded circulating Tregs by 4- to 8-fold (Figure 8K) and led to a tendency toward a reduction in the percentages of CD8^+^ T cells compared with controls (Figure 8L). NK cell frequencies were similar between the mIL-2 and control groups (Figure 8M). Overall, mIL-2 could selectively expand both human and nonhuman primate Tregs in vivo.

## Discussion

In this study, we present the design of an IL-2 mutein (mIL-2) that has a prolonged half-life and selectively expanded Tregs while enhancing their suppressive function. We show the therapeutic efficacy of mIL-2 in 3 murine skin transplant models and the induction of an antigen-specific allograft tolerance that continued after treatment discontinuation. In the most stringent transplant model, some control of the effector arm of the immune system was required to obtain the benefit of Treg expansion from mIL-2 treatment. Last, we demonstrate the Treg selectivity of mIL-2 in human PBMCs and nonhuman primates, laying the foundation for future clinical studies. These findings suggest the utility of in vivo Treg expansion by mIL-2 in transplant recipients to minimize immunosuppression and prolong graft survival.

In vivo Treg expansion by low-dose IL-2 has initially shown clinical success in a few conditions, such as GVHD, hepatitis C virus–induced (HCV-induced) vasculitis, and systemic lupus erythematosus (27–29). More recently, studies investigating low-dose IL-2 in autoimmune diseases have also shown encouraging results (54–56). In transplantation, although low-dose IL-2 or IL-2/anti–IL-2 antibody complexes were capable of prolonging skin transplant (57), pancreatic islet transplant (58, 59), and heart transplant survival in mice (60), clinical studies of low-dose IL-2 demonstrated greater challenges for its translation. In liver transplant recipients, daily subcutaneous injections of low-dose IL-2 (1 × 10^6^ IU/m^2^) expanded circulating Tregs more than 2-fold, but all of the recipients experienced T cell–mediated rejection even before immunosuppression withdrawal (30). This failure was linked to an IFN-γ–orchestrated inflammatory response and the lack of Treg migration into the allografts. In another study, IL-2 at doses of 0.6 × 10^6^ to 3 × 10^6^ IU/m^2^ was administered to nonhuman primates that had undergone a tolerance protocol with bone marrow and kidney transplantation, leading to transient mixed chimerism (61). IL-2 administration led to the activation of effector T cells and precipitated a CD8^+^ T cell–mediated rejection. In vascularized composite tissue transplantation, our group ran a pilot study involving 2 face transplant recipients with the goal of using low-dose IL-2 to promote Tregs and allow minimization of immunosuppression (31). Although low-dose IL-2 led to a robust Treg expansion, significant dose adjustments were required to reduce its off-target effect in NK cells and eosinophils (31). Although one of the patients developed a rejection event after 17 weeks of IL-2 treatment (0.8 × 10^6^ to 2.3 × 10^6^ IU/m^2^, daily), the second patient, who received significantly lower doses of IL-2 every 3 days (0.4 × 10^6^ IU/m^2^), was able to be tapered to tacrolimus monotherapy and completed 1 year of treatment without any rejection events (31). Despite the promising results in the latter patient who received low-dose IL-2, it was clear that a more selective and stable Treg-targeting therapy was needed to translate these findings to the transplant clinic, in the face of IL-2’s narrow therapeutic window. While engineered IL-2 applications have been used in cancer and autoimmunity (62), our study expands this therapeutic strategy to transplantation by showing the efficacy of Treg expansion by a mIL-2 in different transplant models.

Different engineering strategies have been applied to enhance Treg selectivity and prolong the half-life of IL-2, including attaching PEG chains to lysine residues on the surface of the IL-2 molecule, complexing IL-2 with anti–IL-2 antibodies, fusing IL-2 to CD25, or introducing mutations on IL-2 along with coupling with Ig or Fc, among others (37, 63–72). These strategies may increase the stability of the IL-2 molecule and also weaken the interactions of IL-2 with IL-2Rβ and/or IL-2Rγ via destabilization of mutations or epitope masking (64). However, several of these approaches, such as PEGylation, have been shown to weaken the interaction between IL-2 and CD25 (69) and hence the high affinity for CD25/CD122/CD132 receptors on Tregs. Our analysis predicts that such strategies would lead to the activation of alternative cell types, such as NK cells, which have been observed in clinical studies (73) and may counter immunomodulatory activity. We chose the mutein strategy along with the fusion of a human Fc in constructing mIL-2, since it would allow us to maximize the selectivity to Tregs. Other IL-2 muteins like RG7835 and Fc.Mut24 were designed on N88D or N103R and V106D substitutions, respectively, to enhance the Treg selectivity of IL-2 by reducing its interaction with IL-2Rβ (37, 38). In our mIL-2, we sought to maintain WT-like affinity for CD25 while reducing the affinity for CD122/CD132 to curve the pleiotropic affinity of IL-2 away from CD122 while maintaining its CD25 affinity. Given the role of H16 protonation in the IL-2–IL-2R complex recycling that occurs in the endosomes, by modifying this position, we might expect an additional benefit through decreased recycling of CD122 to the cell surface, which can outperform previously identified substitutions in IL-2. RG7835 showed enhanced Treg selectivity in cynomolgus monkeys and humanized mouse models. However, it failed to show efficacy in a phase II trial of ulcerative colitis that was terminated early due to the absence of clinical improvement (NCT03943550). Fc.Mut24 showed higher efficacy compared with Fc–IL-2 in the nonobese diabetic mouse model but still induced proliferation of pancreatic FOXP3^−^CD4^+^ and CD8^+^ T cells that were similar to pancreatic T cells treated with Fc–IL-2 (37). In contrast, our mIL-2 was able to selectively enhance the proliferation of Tregs in the graft-DLN without affecting CD8^+^ T or NK cells, in contrast to WT IL-2 in skin transplant recipients. However, we acknowledge that there may be differences in the efficacy of a certain mIL-2 depending on the disease being tested. As evident in our data, mIL-2 alone cannot prevent a very strong alloimmune response in certain circumstances, such as in a fully mismatched skin transplant model, and requires additional treatments to inhibit the effector T cells simultaneously. We believe our observations are important, as they indicate that drug minimization is a more achievable strategy in highly HLA-mismatched transplants compared with the induction of transplant tolerance.

Tregs exert their suppressive function in secondary lymphoid organs and the transplanted organ (16, 74–76). Although both polyclonal and antigen-specific Tregs can control alloimmunity, donor alloantigen–specific Tregs are significantly more efficient (77–79). Regarding Treg migration to the allograft, low-dose IL-2 failed to promote tolerance in liver transplant recipients and was not associated with increased donor-specific Tregs or trafficking of Tregs to the allograft (30). In contrast, our study of murine skin transplantation revealed that mIL-2 increased graft-infiltrating Tregs and prolonged graft survival. Although the graft-infiltrating Tregs can also be associated with graft rejection as a feedback mechanism, the infiltration in rejection is usually associated with increased CD4^+^ Tconv and CD8^+^ T cells, which were not observed in mIL-2–treated recipients (80, 81). In addition, mIL-2 increased the number of donor-specific Tregs and induced long-term tolerance in a minor-mismatch skin transplant model, even after mIL-2 treatment interruption. The antigen-specific Treg expansion is important, as it provides donor-specific protection to the allograft, which was confirmed by the rejection of third-party grafts (preserved immunity), while the same-donor second grafts survived long term without any treatment. Moreover, mIL-2 decreased antigen-specific CD44^+^CD8^+^ T cells, which have a role in mediating resistance to tolerance induction in transplantation (82). Last, since chronic antibody-mediated rejection is the leading cause of long-term graft loss, the observation of suppression of donor-specific antibody production upon mIL-2 Treg expansion reinforced the possibility that this treatment can improve long-term graft survival.

The suppressive function of Tregs involves myriad mechanisms, including the secretion of immunomodulatory cytokines (e.g., IL-10), downmodulation of DCs, and coinhibitory receptor signaling through CTLA-4 with induction of indoleamine 2, 3-dioxygenase (IDO) (13). In transplantation, the expression of coinhibitory receptors and cytokines such as IL-10 has been demonstrated to be essential for Tregs’ ability to abrogate alloimmune responses (48, 51, 83–85). Equally important to increasing the number of Tregs was ensuring that their function was preserved or enhanced upon mIL-2. Indeed, we showed that mIL-2 increased CTLA-4, ICOS, and LAG3 on Tregs and also their secretion of inhibitory cytokines including IL-10 and LAP. Importantly, we showed that the therapeutic efficacy of mIL-2 was dependent on CTLA-4 (86). Similarly, in an ulcerative colitis model, anti–CTLA-4 antibodies led to the loss of efficacy of adoptively transferred Tregs without affecting the function of Tconv cells (49). In a skin transplant tolerance model, adoptive transfer of Tregs along with Tconv cells led to prolonged graft survival in a CTLA-4–dependent manner, which is also consistent with our data (51). Overall, mIL-2 enhanced the inhibitory markers of Tregs and was associated with greater in vitro suppressive function.

Our study has some limitations. First, we tested mIL-2 only in skin transplant models, which are the most stringent models in transplantation. For extrapolation to other organ transplants, it will be beneficial to test mIL-2 in other situations, in particular solid organ transplant models. Moreover, given its possible application in nontransplant scenarios, future studies should also continue to test mIL-2 in autoimmune diseases. Second, although we showed that an antigen-specific, long-term tolerance could be induced by mIL-2, we have not elucidated the underlying mechanisms, since it is beyond the scope of this study. However, it can be speculated that the tolerance was induced by increasing the number and suppressive capacity of antigen-selective Tregs and by suppression of antigen-specific effector T and B cells. Third, we showed that the effects of mIL-2 were CTLA-4 dependent. Although the previous studies and our data show that the anti–CTLA-4 antibodies we used in our experiments are non-Treg-depleting (49, 51–53), we observed a loss of Treg expansion after day 7. This was likely due to the loss of Treg proliferation with the unavailability of CTLA-4.

In conclusion, we present an IL-2 mutein that is optimized with an enhanced Treg selectivity and a prolonged half-life and demonstrate its efficacy in skin transplant models. The findings of our study indicate the utility of in vivo Treg expansion in transplantation to promote antigen-specific peripheral immune regulation and allow immunosuppression minimization. This approach is particularly helpful in view of the known detrimental effects of current immunosuppressive drugs on Tregs (15, 57, 87, 88). By showing the selective and sustainable Treg expansion in human and nonhuman primate models, we set the stage for clinical testing of mIL-2 in transplant recipients.

## Methods

### Sex as a biological variable.

Our study examined male and female animals, and similar findings are reported for both sexes.

### DNA and proteins.

DNA sequences encoding all proteins were synthesized at Twist Bioscience in pcDNA3.1 (Thermo Fisher Scientific) and transfected into Expi293 cells using ExpiFectamine (Thermo Fisher Scientific), as described by the manufacturer. Fc–IL-2 fusion protein (G_4_S)_4_ linkers, with or without a C-terminal AviTag, were expressed in HEK293T cells and purified using protein A chromatography. To create a CD122/CD132 stable dimer, a knob-hole format was used with the extracellular domain of CD122 (GenBank: KAI2597668.1) fused through a (G_4_S)_2_ linker to knob human IgG1 Fc (N297G, S354C, T366W) and the extracellular domain of CD132 (GenBank: NP_000197.1) through a (G_4_S)_2_ linker to the hole human IgG1 Fc (N297G, Y349C, T366S, L368A, Y407V). The Fc mutations were made to preference heterodimer formation over the formation of homodimers (so-called “knobs-in-hole”) (89). Plasmids were cotransfected, and CD122/CD132 dimer was purified from the supernatant using protein A chromatography. The ectodomain of recombinant human CD25 was obtained from R&D Systems. WT Fc–IL-2 differed from mIL-2 solely at amino acid position 16. AviTag Fc–IL-2 fusion proteins were site-specifically biotinylated using the BirA enzyme as described by the manufacturer (BPS Bioscience).

### Yeast display.

Yeast surface display was performed as previously described (90). Briefly, the human IL-2 sequence (GenBank: AAB46883.1) was modified using site saturation mutagenesis, error-prone PCR, or synthesis of sequences with directed substitutions. Amplicons were cotransformed with a linearized expression vector into EBY100 yeast and cultured as previously described (91). Yeasts expressing the IL-2 variants were assessed in 2 distinct ways. To measure expression levels (a surrogate for aggregation and stability), yeast were stained with fluorescent antibodies against the c-Myc epitope tag present at the C-terminus of the IL-2 protein. Additionally, yeasts were incubated with a recombinant CD122/CD132 heterodimer, and binding was detected with goat anti–human Fc conjugated with a fluorophore. Yeasts expressing high levels of IL-2 and/or reduced binding to CD122/CD132 were sorted using a Sony MA900 flow cytometer. The *IL2* gene sequences within the enriched libraries were PCR amplified and sequenced with an Illumina MiSeq 2 × 75 PE (Genewiz).

### Binding analysis.

All binding assessments were performed using an Octet RED384 (Sartorius). For CD25 binding experiments, Fc–IL-2 constructs were captured with anti–human IgG Fc (AHC) Biosensors (Sartorius), and recombinant human CD25 was added to measure binding. For CD122/CD132 binding assessment, biotinylated Fc–IL-2 constructs were captured on streptavidin (SA) biosensors and CD122/CD132-Fc heterodimer was added to assess binding kinetics.

### Mice.

FOXP3-GFP-B6 (C57BL/6-Tg(*FOXP3-*GFP)90Pkraj/J) and B6.mOVA mice (C57BL/6-Tg(*CAG-OVAL*)916Jen/J (92) were obtained from breeding colonies at Mass General and Brigham facilities. B6 (C57BL/6J), BALB/c (BALB/cJ), NSG (NOD.Cg-*Prkdc^scid^ Il2rg^tm1Wjl^*/SzJ), and transgenic Tg32 (B6. Cg-*Fcgrt* Tg(*FCGRT*)32Dcr/DcrJ mice expressing human FcRn) mice were purchased from The Jackson Laboratory. All mice were used at 6–10 weeks of age with an average weight of 18–20 g.

### Pharmacokinetics assessment of mIL-2 in Tg32 mouse.

Transgenic Tg32 mice lack mouse FcRn and express human FcRn under the control of its native promoter. Tg32 mice were administered test proteins intravenously, and blood samples were obtained from all animals at hours 1, 6, 12, 24, 72, 120, and 168 following administrations. Antibodies and Fc–IL-2 fusion proteins were quantified from mouse serum using an ELISA specific for human IgG1 Fc and IL-2 (R&D Systems, AF-202).

### Skin transplantation models.

Three mouse skin transplant models were used: B6.mOVA to WT B6, male to female B6, and BALB/c to B6. Approximately 1 cm^2^ of full-thickness tail skin was collected from donor mice and transplanted onto the dorsum of recipient mice via suturing. Mice were anesthetized with isoflurane inhalation. Postoperative analgesia was achieved with buprenorphine (PAR Pharmaceutical). Grafts were secured with an adhesive strip for 7 days. Graft rejection was diagnosed when less than 10% of the graft remained viable. For graft tolerance experiments, a similar alloskin and a third-party skin graft were transplanted at the dorsal right and left of the original graft. For treatments, recipient mice were given subcutaneous injections of control human IgG, Fc–IL-2, or mIL-2 at the indicated doses and times. Tacrolimus (Astellas) was given intraperitoneally at 5 mg/kg daily starting 3 days before transplantation. In mechanistic experiments, 2 anti–CTLA-4 antibodies (Bio X Cell, clones UC10-4F10-11 and 9H10) were given intraperitoneally at 200 μg/mouse for each, twice a week starting on day 0.

### NSG expansion model.

PBMCs were isolated from healthy volunteers using Ficoll density-gradient centrifugation and washed with 1× PBS. Twenty million PBMCs per mouse were transferred into 8-week-old NSG mice via retro-orbital injection. Fc–IL-2 or mIL-2 were given subcutaneously on days 0 and 4, and spleens were harvested on day 7 for flow cytometry. Mice were pre-randomized for the study groups.

### Nonhuman primates.

Cynomolgus monkeys were administered 0.5 mg/kg mIL-2 or PBS subcutaneously on days 0, 7, and 14. Animals were bled on days 0, 2, 4, 7, 14, and 21. PBMCs were assessed by flow cytometry for expression of CD45 (PerCP, BD Biosciences, catalog 558411), CD3 (APC-Cy7, BD Biosciences, catalog 557757), CD4 (FITC, BD Biosciences, catalog 550628), CD8 (BV510, BioLegend, catalog 344732), CD20 (BV421, BioLegend, catalog 302330), CD25 (PerCP-Cy5.5, BioLegend, catalog 302626), CD159a (PE-Cy7, Coulter, catalog B10246), and FOXP3 (PE, BioLegend, catalog 320108).

### In vitro stimulation of splenocytes.

Cells were stimulated with PMA (10 ng/mL), ionomycin (0.5 μM), and brefeldin A (3 μg/mL) cocktail for 4–6 hours for assessment of cytokine secretions. After stimulation, cells were stained extracellularly with the viability dye DAPI (Invitrogen, Thermo Fisher Scientific), CD3 (17A2, APC/Cyanine 7), CD4 (RM4-5, Brilliant Violet 510), CD8 (53-6.7, Alexa Fluor 700), CD25 (PC61, PE/Dazzle 594), CD19 (6D5, Pacific Blue), CD11b (M1/70, Pacific Blue), CD11c (N418, Pacific Blue), NK1.1 (PK136, Pacific Blue), and GR1 (RB6-8C5, Pacific Blue) and intracellularly with FOXP3 (MF14, Alexa Fluor 488), LAP (S20006A, APC), IL-10 (JES3-9D7, PE/Cyanine7), and IFN-γ (XMG1.2, Brilliant Violet 605) (all BioLegend).

### Treg suppression assay.

Splenic Tregs (CD4^+^FOXP3^+^) from mIL-2– or control IgG–treated B6 FOXP3-GFP mice and Tconv (CD4^+^FOXP3^–^) cells from naive B6 mice were sorted. Tconv cells were stained with 1 μM Violet Proliferation Dye (VPD450, Thermo Fisher Scientific) for 25 minutes at 37°C, followed by a 5-minute recovery in complete media at 37°C. After being washed twice, Tconv cells were cocultured with Tregs at different ratios in the presence of anti–mouse CD3/CD28-conjugated beads (Thermo Fisher Scientific) at 37°C for 72 hours. Cells were stained with viability dye (7-amino-actinomycin D, BioLegend), and proliferation was assessed by flow cytometry. The percentage of suppression was determined by calculating the ratios as follows: (Tconv proliferation without Treg – Tconv proliferation with Tregs)/(Tconv proliferation without Tregs).

### Donor-specific antibodies.

For anti-OVA antibodies, MaxiSorp plates were coated with 100 μg/well OVA (InvivoGen) by incubation overnight at 4°C. Plates were blocked with 1% BSA in PBS-T for 1 hour at room temperature. Serial dilutions of control antibodies and mice sera in a dilution of 1:3,000 were added and incubated at 37°C for 3 hours. Samples were incubated with secondary anti–mouse IgG antibodies with peroxidase followed by the addition of TMB (3,3′,5,5′–tetramethylbenzidine). After adding the stop solution, plates were read on a Spectramax plate reader at 450 nm.

### In vitro p-STAT5 assay.

Splenocytes were isolated from B6 FOXP3-GFP mice and resuspended in RPMI 10% FBS. Human PBMCs were isolated from the peripheral blood of healthy volunteers. One million cells per well were plated into a 96-well, U-bottomed plate and treated with increasing concentrations of control IgG, mIL-2, or Fc–IL-2 for 30 minutes at 37°C. Cells were fixed with 10% PFA in PBS for 10 minutes at room temperature and permeabilized with TruPhos buffer (BioLegend) (–20°C) for 5 minutes. Cells were stained with both surface and intracellular antibodies including anti–p-STAT5 [clone 47/STAT5 (pY694), Pacific Blue, BD Biosciences] simultaneously at room temperature for 30 minutes.

### Tetramer staining.

MHC class I and II tetramers were manufactured by the NIH tetramer core. B6 MHC class I (H2-K^b^) tetramers were developed using OVA_257–264_ (SIINFEKL) peptide, which was conjugated to phycoerythrin (PE). For MHC II, OVA_329–337_:I-A^b^ (AAHAEINEA) and OVA_325–335_:I-A^b^ (QAVHAAHAEIN) tetramers were used in conjugation with PE. To increase the visibility of antigen-specific T cells, pooled reagents of the 2 MHC class II tetramers were used together. Staining was performed at 37°C for 30 minutes prior to surface and intracellular staining.

### Histopathology.

Skin grafts were fixed in formalin for 24–48 hours at 4°C and then stored in 70% ethanol at room temperature. The tissues were embedded in paraffin, sectioned at 5 μM thickness, and stained with H&E for morphological analysis. IHC staining was performed with an automated Ventana BenchMark Stain System (Roche) at MGH Pathology Core Laboratories using mouse antibodies against CD3, FOXP3, and CD31. Stainings were confirmed with negative and positive controls.

### Flow cytometry.

For only-surface staining, whole blood was mixed with antibodies at room temperature for 30 minutes, and BD FACS lysis buffer was added for RBC lysis. When both surface and intracellular staining were required, RBCs were first lysed with ACK buffer and then stained with surface and intracellular antibodies using a fixation/permeabilization kit (Thermo Fisher Scientific). For in vivo mouse experiments, the following anti-mouse antibodies were used: CD45 (30-F11, Alexa Fluor 700), CD3 (17A2, APC/Cyanine 7), CD4 (GK1.5, PerCP/Cyanine5.5), CD19 (1D3/CD19, Pacific Blue), CD8 (53-6.7, PE/Cyanine7), CD4 (OKT4, PerCP/Cyanine5.5), FOXP3 (MF-14, Alexa Fluor 488), CD25 (PC61, APC), NK1.1 (PK136, PerCP/Cyanine5.5), Siglec-F (S17007L, PE), CD11b (M1/70, APC), GR1 (RB6-8C5, PE/Cyanine 7), CD11c (N418, Pacific Blue), F4/80 (BM8, Pacific Blue), and Ter119 (TER119, Pacific Blue) (all BioLegend). For human PBMC staining, the following anti-human antibodies were used: CD45 (2D1, Alexa Fluor 700), CD3 (OKT3, FITC), CD19 (SJ25C1, Brilliant Violet 785), CD8 (SK1, PE/Cyanine7), CD4 (RPA-T4, APC/Cyanine7), CD25 (BC96, PE/Dazzle 594), FOXP3 (206D, Pacific Blue), CD127 (A019D5, PerCP/Cyanine5.5), CD56 (QA17A16, PE/Cyanine7), CD15 (W6D3, Brilliant Violet 711), and CD16 (3G8, FITC) (all from BioLegend). Stained cells were analyzed on a FACSCanto II flow cytometer (BD Biosciences) with FACSDiva software (BD Biosciences). Data were analyzed with FlowJo software (TreeStar). the gating strategies used are described in Supplemental Figures 7–9.

### Study approval.

Informed consent was obtained for blood collection from healthy human donors. All animals were housed and experiments were conducted with the approval of the IACUC of Massachusetts General Hospital (Boston, Massachusetts, USA) and followed NIH animal care guidelines (protocols 2020N000085 and 2020N000149).

### Statistics.

For descriptive statistics, the means with SD were used for continuous variables, and percentiles were used for categorical variables. For 2-group comparisons, a 2-tailed simple or paired *t* test was used. For comparisons of more than 2 groups, 1-or 2-way ANOVA was used followed by Tukey’s multiple-comparison test for post hoc analysis. Skin allograft survival was calculated using the Kaplan-Meier method and compared between the groups using the log-rank test. A *P* value of less than 0.05 was considered statistically significant. Data were analyzed with GraphPad Prism 5.0 (GraphPad Software).

### Data availability.

Values for all data points in graphs are reported in the Supplemental Supporting Data Values file.

## Author contributions

LVR, OE, TJB, and GJB planned the study, interpreted the results, and wrote the manuscript. ZS and GJB, designed mIL-2, supplied material for the study, and provided the data from the nonhuman primate experiments. OE, TJB, RBG, LM, YG, AAJ, ITL, and PVA conducted the mouse and in vitro experiments, helped with selection of the mIL-2 variants, and analyzed the data. JCM and CL helped to interpret the results and edited the manuscript.

## Supplementary Material

Supplemental data

Supporting data values

## Figures and Tables

**Table 1:**
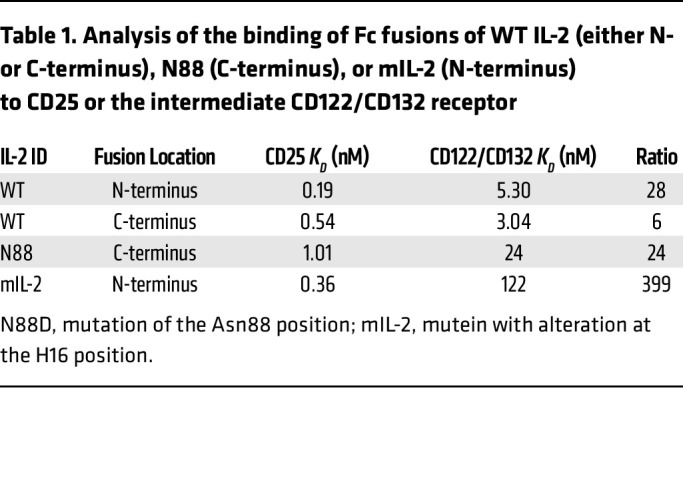
Analysis of the binding of Fc fusions of WT IL-2 (either N- or C-terminus), N88 (C-terminus), or mIL-2 (N-terminus) to CD25 or the intermediate CD122/CD132 receptor

## Version 1 03/01/2024

Electronic publication
