# Supplementary material for: A humanized IL-2 mutein expands Tregs and prolongs transplant survival in preclinical models
Source: J Clin Invest. 2024 Mar 1;134(5):e173107. doi: 10.1172/JCI173107 (PMC10904054; doi:10.1172/JCI173107)
Supplement: Supplemental data [file jci-134-173107-s080.pdf]

A humanized IL-2 mutein expands Tregs and prolongs transplant survival in preclinical models

Supplementary Materials

1- Supplementary Figures

- Figure S1. Comparison of subcutaneous and intravenous administration of mIL-2 in vivo.
- Figure S2. Blood and draining lymph node flow cytometry and plasma anti-OVA antibodies in B6.mOVA to wild-type skin transplantation
- Figure S3. Effect of mutein IL-2 in male to female minor mismatch B6 skin transplantation
- Figure S4. Long-term sustainability of Treg expansion by mutein IL-2 in skin transplant recipient mice and its wearing off post-treatment discontinuation
- Figure S5. Effect of tacrolimus and control IgG combination in BALB/c to B6 skin transplant model
- Figure S6. Effect of mutein IL-2 and CTLA-4 Ig combination in BALB/c to B6 skin transplant model
- Figure S7. Flow cytometry gating strategy in mouse splenocyte pSTAT5 assay and ex vivo male to female B6 mouse skin transplant model
- Figure S8. Flow cytometry gating strategy in ex vivo experiments in B6.mOVA to wild type B6 and BALB/c to B6 mouse skin transplant models
- Figure S9. Flow cytometry gating strategy of human PBMCs in pSTAT5 assay and humanized NSG mouse model.

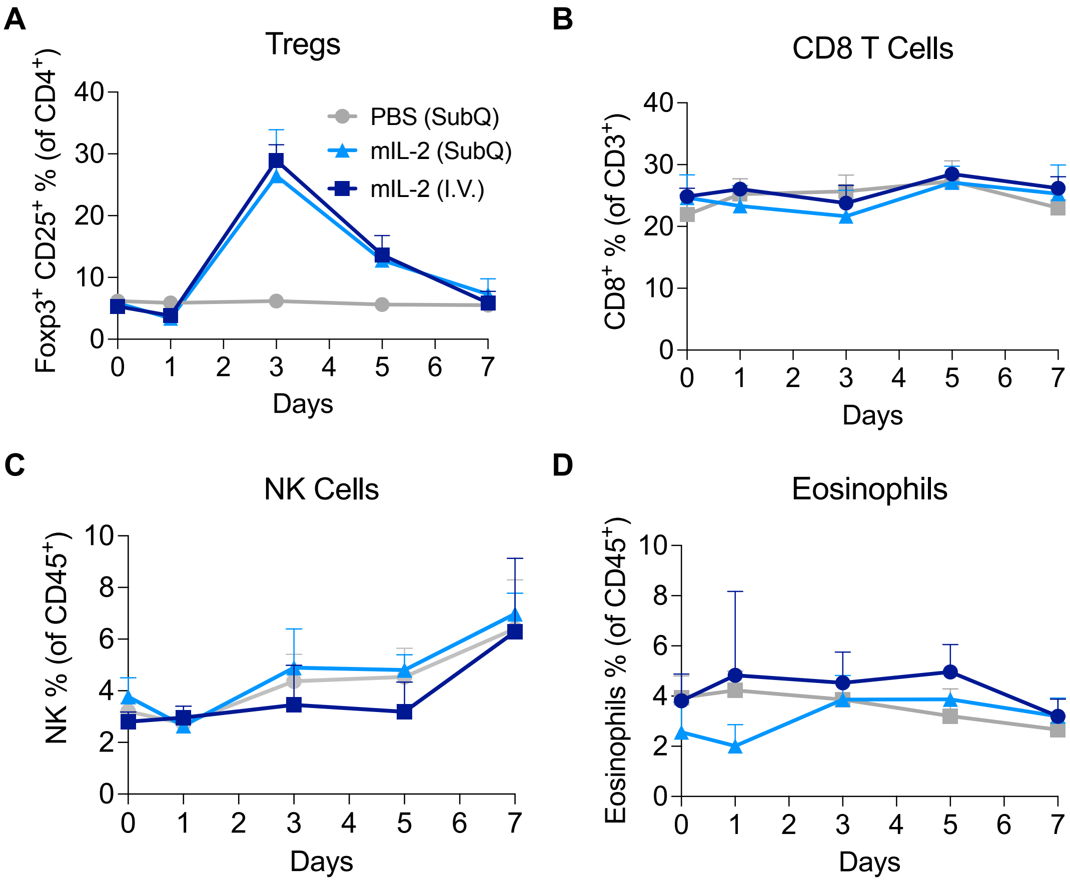

Figure S1. Comparison of subcutaneous and intravenous administration of mIL-2 *in vivo*. Flow cytometry analysis of circulating (**A**) Tregs, (**B**) $CD8^+$ T cells, (**C**) NK cells, and (**D**) eosinophils following a single administration of subcutaneous or intravenous mIL-2 at 0.5 mg/kg/mouse compared to subcutaneous injection of vehicle (PBS), (n = 3/group, data from single experiment). Graphs are displayed as mean ± SD and one-way ANOVA with Tukey's multiple comparisons test is used for the group comparisons.

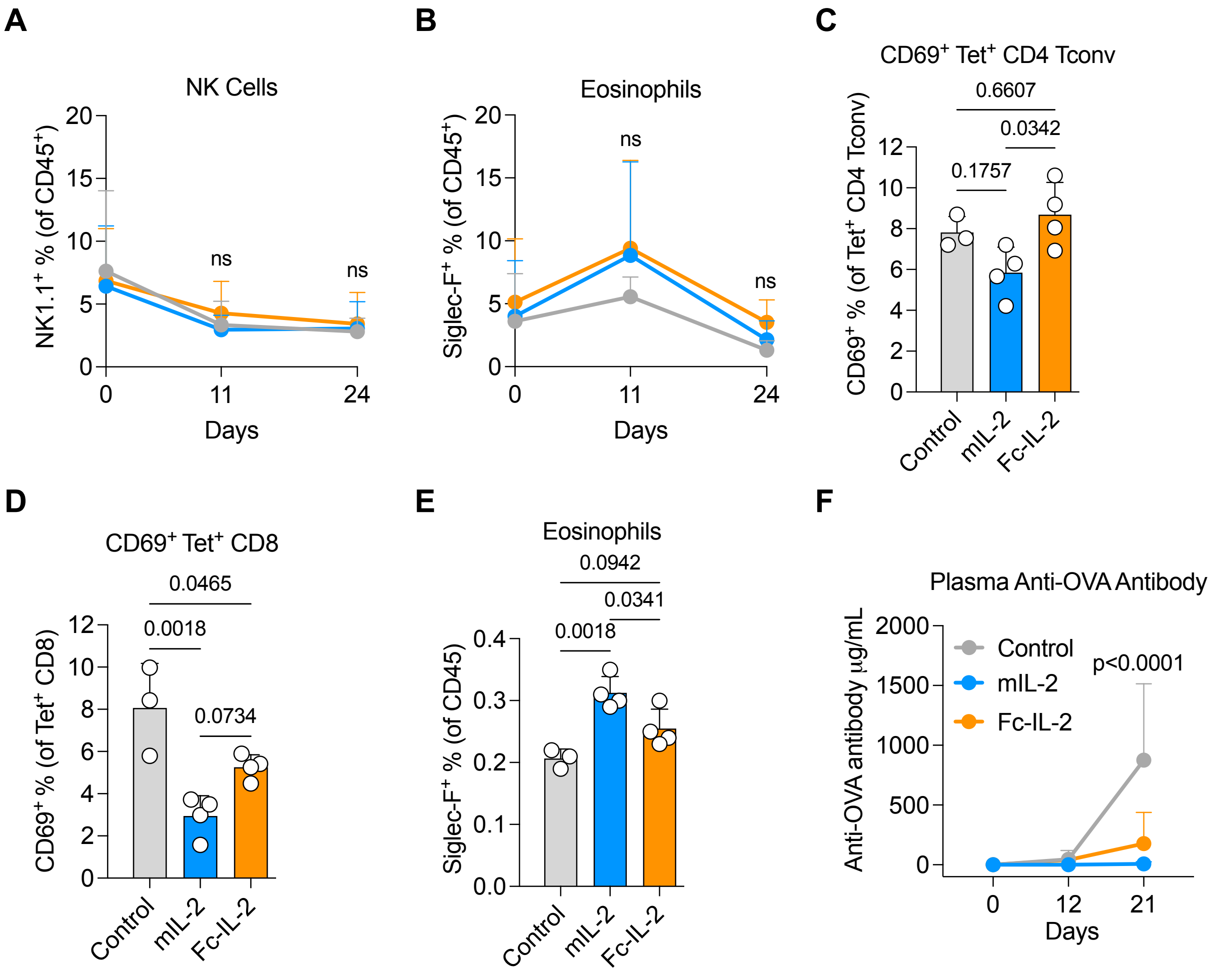

Figure S2. Blood and draining lymph node flow cytometry and plasma anti-OVA antibodies in B6.mOVA to wild-type skin transplantation. Blood flow cytometry analysis showing the frequencies of (**A**) NK cells and (**B**) eosinophils (n = 4-9/per group, data pooled from 3 independent experiments). Flow cytometry analysis of the graft DLN at day 12 showing the frequencies of (**C**) CD69^{+} tetramer^{+} CD4^{+} Tconv, (**D**) CD69^{+} tetramer^{+} CD8^{+} T cells, and (**E**) eosinophils (n = 4/group). (**F**) Plasma anti-OVA antibody levels at days 0, 12, and 21 (n = 4-8/group, data pooled from 3 independent experiments). Graphs are displayed as mean ± SD and one-way and two-way ANOVA with Tukey's multiple comparisons tests are used for the group comparisons as appropriate.

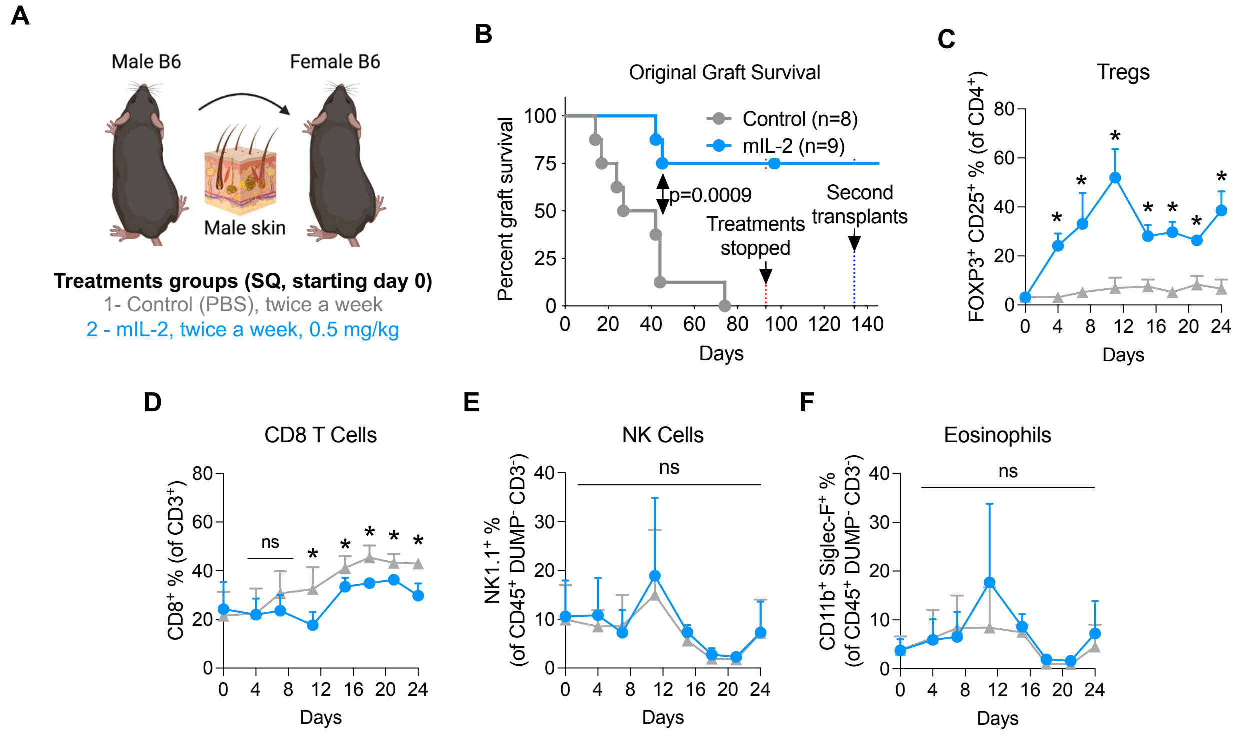

Figure S3. Effect of mutein IL-2 in male to female minor mismatch B6 skin transplantation. (**A**) An illustration of the experiment design and (**B**) Kaplan Meier graph of the graft survival in male to female B6 mouse skin transplantation where the recipient mice were treated with mIL-2 at 0.5 mg/kg or vehicle (PBS) subcutaneously twice a week starting at day 0. Flow cytometry analysis of peripheral blood showing the frequencies of (**C**) Tregs, (**D**) $CD8^+$ T cells, (**E**) NK cells, and (**F**) eosinophils between days 0 and 21 (n = 8-9/group, data pooled from 2 independent experiments). Graphs are displayed as mean ± SD and a t-test is used for the group comparisons (**C-F**). The long-rank test is used for graft survival comparisons (**B**) (* = p≤0.05; ns = non-significant (p>0.05)).

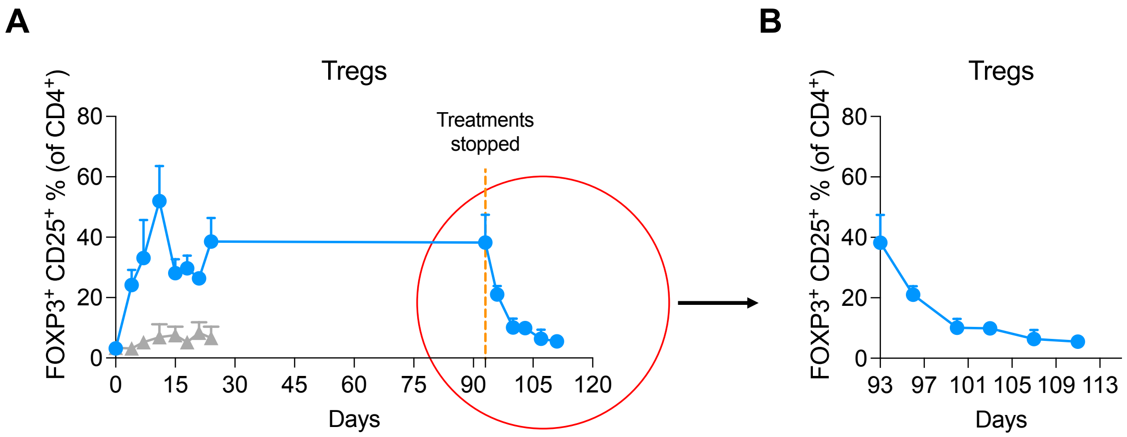

Figure S4. Long-term sustainability of Treg expansion by mutein IL-2 in skin transplant recipient mice and its wearing off post-treatment discontinuation. (**A**) Flow cytometry analysis of peripheral blood showing the stability of Treg expansion until the treatments were stopped at median day 93 and subsequent decrease in Treg levels returning to normal within 2-3 weeks in male to female skin transplantation model. (**B**) A magnified version of the Treg percentiles between days 93-111. Graphs are displayed as mean ± SD.

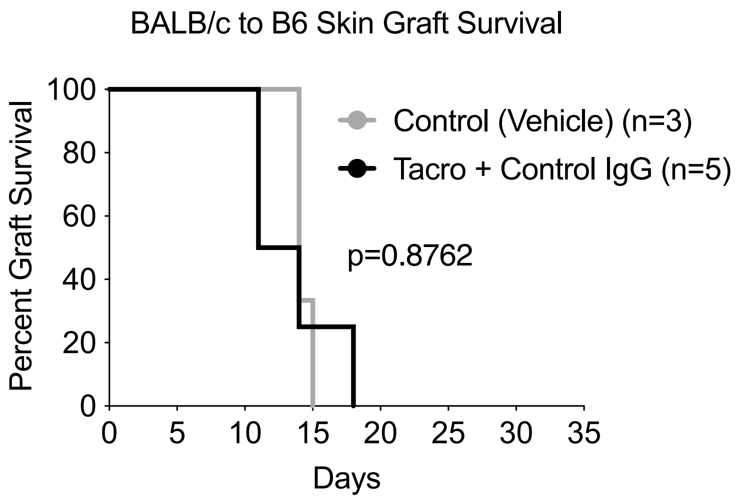

Figure S5. Effect of tacrolimus and control IgG combination in BALB/c to B6 skin transplant model. Kaplan Meier survival curve comparing the skin graft survival in control (vehicle) and tacrolimus + control IgG treated BALB/c to B6 skin transplant recipient mice. The long-rank test is used for graft survival comparison.

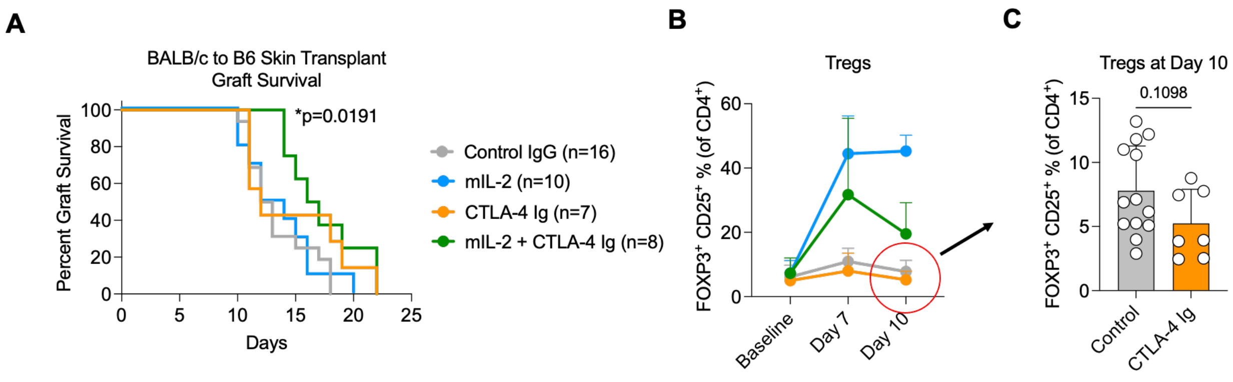

Figure S6. Effect of mutein IL-2 and CTLA-4 Ig combination in BALB/c to B6 skin transplant model. (**A**) Kaplan Meier graph of the graft survival in BALB/c to B6 mouse skin transplantation model where the recipient mice were treated with subcutaneous mIL-2 at 0.5 mg/kg twice a week starting at day 0 and/or intraperitoneal CTLA-4 Ig at 500 µg/mouse on days 0, 2, 4, and 6 or vehicle treatments. (**B**) Flow cytometry analysis of peripheral blood showing the frequencies of circulating Tregs at baseline and days 7 and 10. (**C**) A magnified version of peripheral Treg comparison at day 10 in control vs. CTLA-4 Ig groups (n = 7-16 /group, data pooled from 4 independent experiments). Graphs are displayed as mean ± SD and a two-way ANOVA with Tukey's multiple comparisons test is used for >3 group comparisons (**B**) and a t-test for 2 group comparisons (**C**). The long-rank test is used for graft survival comparisons (**A**).

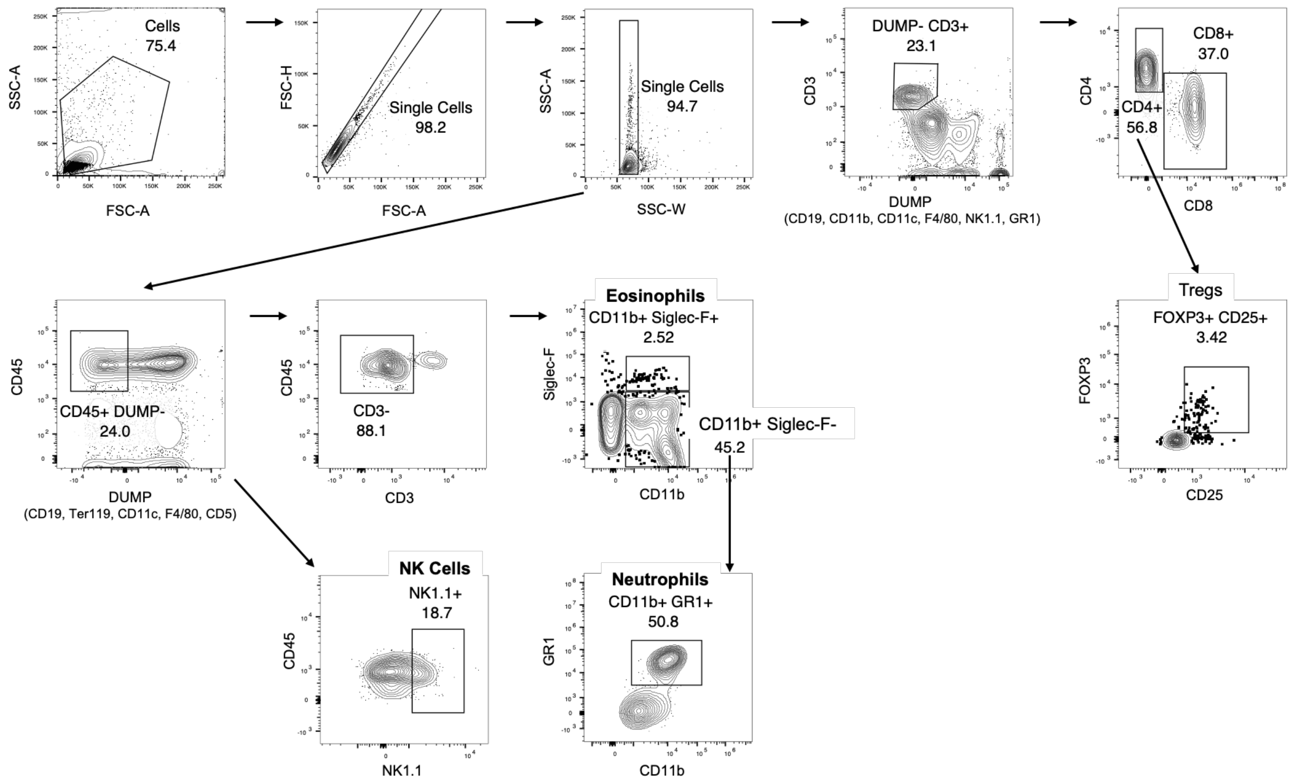

Figure S7. Flow cytometry gating strategy in mouse splenocyte pSTAT5 assay and *ex vivo* male to female B6 mouse skin transplant model.

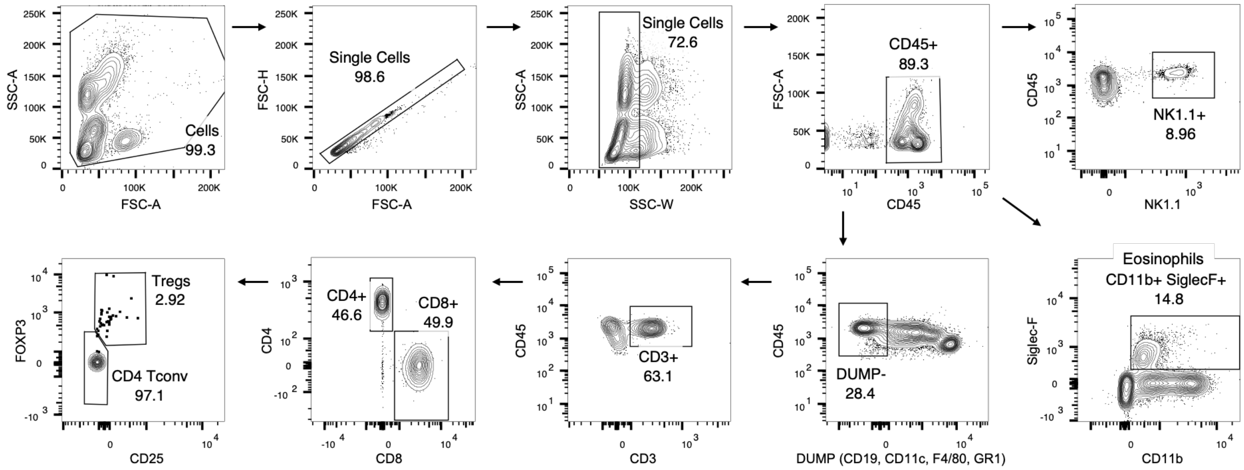

Figure S8. Flow cytometry gating strategy in *ex vivo* experiments in B6.mOVA to wild type B6 and BALB/c to B6 mouse skin transplant models.

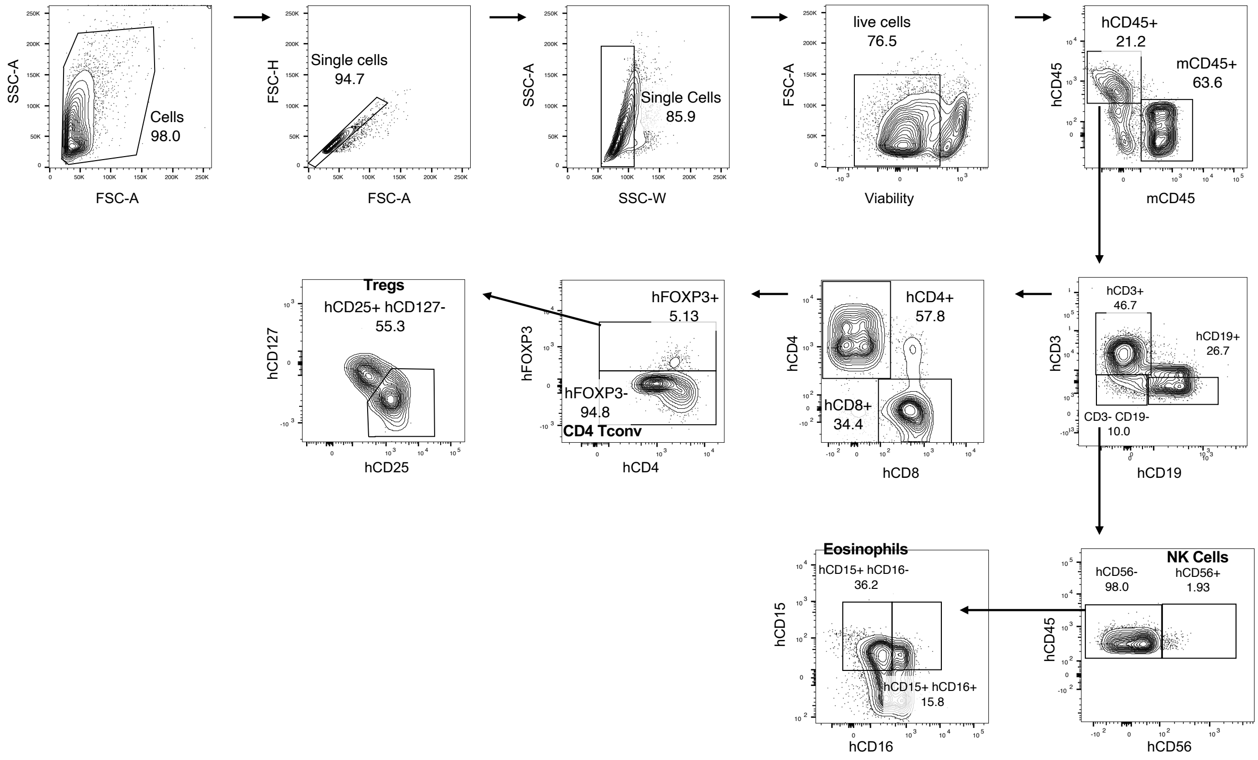

Figure S9. Flow cytometry gating strategy of human PBMCs in pSTAT5 assay and humanized NSG mouse model.
